# Mint3 depletion restricts tumor malignancy of pancreatic cancer cells by decreasing SKP2 expression via HIF-1

**DOI:** 10.1038/s41388-020-01423-8

**Published:** 2020-08-21

**Authors:** Akane Kanamori, Daisuke Matsubara, Yurika Saitoh, Yuya Fukui, Noriko Gotoh, Shuichi Kaneko, Motoharu Seiki, Yoshinori Murakami, Jun-ichiro Inoue, Takeharu Sakamoto

**Affiliations:** 1 Division of Cellular and Molecular Biology, the Institute of Medical Science, The University of Tokyo, Shirokanedai, Minato-ku, Tokyo Japan; 2 Departments of Pathology, Jichi Medical University, Yakushiji, Shimotsuke-shi, Tochigi Japan; 3 Center for Medical Education, Teikyo University of Science, Senjusakuragi, Adachi-ku, Tokyo Japan; 4 Division of Cancer Cell Biology, Cancer Research Institute, Kanazawa University, Kakuma-machi, Kanazawa, Ishikawa Japan; 5 Department of Gastroenterology, Institute of Medical, Pharmaceutical and Health Sciences, Kanazawa University, Takaramachi, Kanazawa, Ishikawa Japan; 6 Division of Cancer Cell Research, the Institute of Medical Science, The University of Tokyo, Shirokanedai, Minato-ku, Tokyo Japan; 7 Division of Molecular Pathology, the Institute of Medical Science, The University of Tokyo, Shirokanedai, Minato-ku, Tokyo Japan; 8 Department of System Biology, Institute of Medical, Pharmaceutical and Health Sciences, Kanazawa University, Takaramachi, Kanazawa, Ishikawa Japan

**Keywords:** Pancreatic cancer, Cell growth, Transcription

## Abstract

Pancreatic cancer is one of the most fatal cancers without druggable molecular targets. Hypoxia inducible factor-1 (HIF-1) is a heterodimeric transcriptional factor that promotes malignancy in various cancers including pancreatic cancer. Herein, we found that HIF-1 is accumulated in normoxic or moderate hypoxic areas of pancreatic cancer xenografts in vivo and is active even during normoxia in pancreatic cancer cells in vitro. This prompted us to analyze whether the HIF-1 activator Mint3 contributes to malignant features of pancreatic cancer. Mint3 depletion by shRNAs attenuated HIF-1 activity during normoxia and cell proliferation concomitantly with accumulated p21 and p27 protein in pancreatic cancer cells. Further analyses revealed that Mint3 increased transcription of the oncogenic ubiquitin ligase SKP2 in pancreatic cancer cells via HIF-1. This Mint3-HIF-1-SKP2 axis also promoted partial epithelial-mesenchymal transition, stemness features, and chemoresistance in pancreatic cancer cells. Even in vivo, Mint3 depletion attenuated tumor growth of orthotopically inoculated human pancreatic cancer AsPC-1 cells. Database and tissue microarray analyses showed that Mint3 expression is correlated with SKP2 expression in human pancreatic cancer specimens and high Mint3 expression is correlated with poor prognosis of pancreatic cancer patients. Thus, targeting Mint3 may be useful for attenuating the malignant features of pancreatic cancer.

## Introduction

Pancreatic ductal adenocarcinoma is a highly aggressive and lethal tumor, characterized by invasiveness, rapid progression, and treatment resistance [1, 2]. Despite numerous studies revealing the molecular and genetic alterations of pancreatic ductal adenocarcinoma, the recent 5-year survival rate is still ~9% [3]. Due to the lack of druggable molecular targets, cytotoxic chemotherapy drugs such as gemcitabine and nab-paclitaxel are mainly applied for pancreatic ductal adenocarcinoma treatment with insufficient outcomes. Therefore, effective molecular targeting drugs need to be developed to combat pancreatic cancer.

Hypoxia inducible factor-1 (HIF-1) is a heterodimeric transcriptional factor that adapts to low oxygen to maintain tissue homeostasis and is comprised of an oxygen-sensitive α subunit and a constitutive β subunit. The α subunit of HIF-1 is suppressed under normoxic conditions by two types of oxygen-dependent hydroxylases, HIF prolyl hydroxylase domain containing proteins (PHDs) and factor inhibiting HIF-1 (FIH-1) [4–7]. PHDs hydroxylate the proline residues of HIF-1α, inducing its proteasomal degradation. In turn, FIH-1 hydroxylates the asparagine residues of HIF-1α, inhibiting interactions between HIF-1α and transcriptional co-factor p300/CBP and resulting in its transcriptional inactivation.

Hypoxia is a characteristic of tumors including those of pancreatic cancer and the functions of HIF-1 during severe hypoxia have been extensively studied. Interestingly, only 50–60% of solid tumors exhibit hypoxic areas [8], and HIF-1α protein is instead decreased in severe hypoxic areas due to tumor necrosis in patients with cervical cancer and xenografted tumors [9]. Therefore, the pathophysiological roles of HIF-1 in the presence of sufficient oxygen in cancer cells are of particular interest. Oncogenic signaling pathways such as the Ras/Raf/MEK/ERK pathway and the PI3K/AKT/mTOR pathway are known to increase HIF-1α protein during normoxia by enhancing transcription and translation of HIF-1α in cancer cells [7, 10]. However, increased HIF-1α proteins must further evade suppression by FIH-1 to fully function as a transcriptional factor during normoxia.

Munc18-1-interacting protein 3 (Mint3) can activate HIF-1 even under normoxic conditions by binding to and suppressing FIH-1 without affecting protein levels of HIF-1α [11]. Mint3 itself is expressed ubiquitously, but Mint3-mediated HIF-1 activation is limited to some types of cells, such as cancer cells, macrophages, and cancer-associated fibroblasts, mainly due to the necessity of matrix metalloproteinase 14 expression, which supports stable interactions between Mint3 and FIH-1 in cells [7, 12–16]. Previously, we revealed that Mint3 depletion reduces HIF-1 activity during normoxia and tumorigenicity in breast cancer and fibrosarcoma [15, 17]. However, whether and how Mint3 contributes to HIF-1 activation during normoxia and tumor malignancy in pancreatic cancer cells remain unclear.

Here, we addressed whether Mint3-mediated HIF-1 activity during normoxia contributes to the malignant features of pancreatic cancer cells by employing Mint3-depleted pancreatic cancer cells. We found that Mint3 depletion decreased the expression of the S-phase kinase associated protein (SKP2) oncogene [18] via HIF-1 during normoxia, thereby attenuating malignant features such as cell proliferation, stemness, chemoresistance, and tumorigenicity in pancreatic cancer cells.

## Results

### Mint3 depletion attenuates cell proliferation in pancreatic cancer cells

**Fig. 1:**
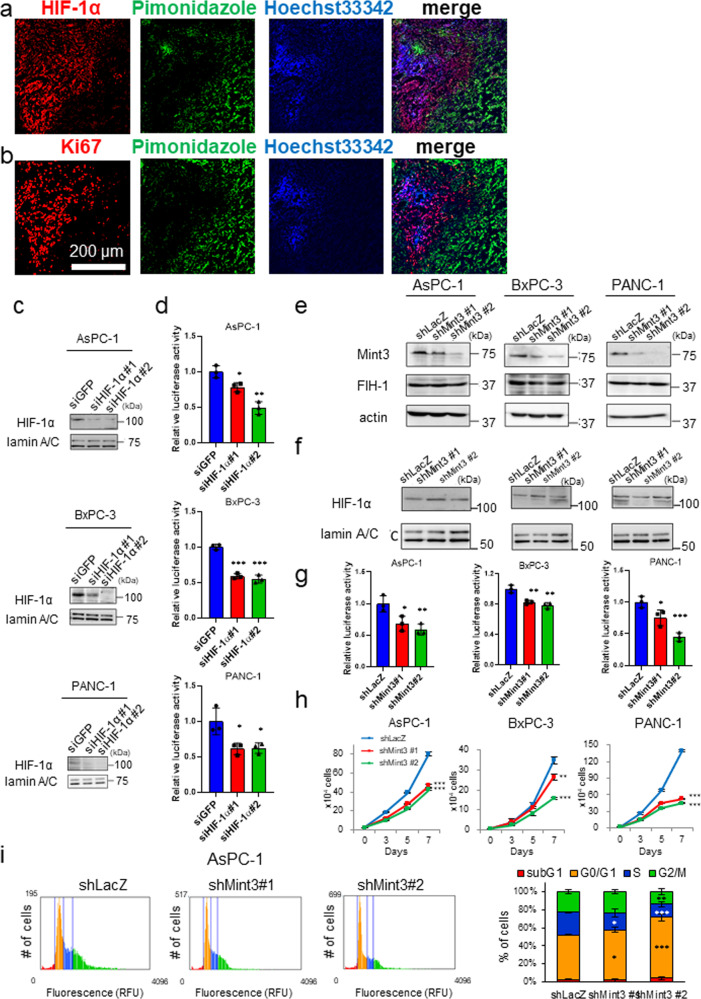
Mint3 knockdown decreases cell growth and induces cell cycle arrest in pancreatic cancer cells. Immunostaining of hypoxic marker pimonidazole (green) and HIF-1α (**a**) or Ki67 (**b**) (red) in pancreatic xenografts of AsPC-1 cells. Nuclei of cells around near blood vessels were counterstained with Hoechst 33342 (blue). **c** Immunoblotting of HIF-1α and lamin A/C in nuclear extracts from control (shLacZ) and HIF-1α-depleted (shHIF-1α) pancreatic cancer cells. **d** HIF-1 activity in control and HIF-1α-depleted pancreatic cancer cells. **e** Mint3, FIH-1, and actin expression in control (shLacZ) and Mint3-depleted (shMint3) pancreatic cancer cells. **f** HIF-1α and lamin A/C expression in nuclear extracts from control and Mint3-depleted pancreatic cancer cells. **g** HIF-1 activity in control and Mint3-depleted pancreatic cancer cells. **h** Cell growth assay of pancreatic cancer cells. **i** Cell cycle analysis of control and Mint3-depleted AsPC-1 cells. Representative graphs of propidium iodide staining (left). The distribution of each cell cycle phase in AsPC-1 cells (right). Error bars indicate SD (*n* = 3). *p < 0.05, **p < 0.01, ***p < 0.001 (*t*-test).

Although HIF-1 is known to be upregulated during hypoxia [8], HIF-1 protein expression is reduced in most hypoxic regions of tumor tissues from cervical cancer clinical specimens and xenografts due to necrosis [9]. To assess whether this HIF-1α protein localization in normoxic and mild hypoxic areas is also observed in pancreatic cancer xenografts, we performed immunostaining of HIF-1α protein and pimonidazole, which forms adducts of thiol-containing proteins in cells with <10 mm Hg of pO_2_ [19] in tumors of orthotopically injected human pancreatic cancer AsPC-1 cells that were also perfused with Hoechst 33342 via intravenous injection to detect cells near functional blood vessels. The majority of HIF-1α proteins were found localized outside pimonidazole-positive hypoxic regions, where most cells were labeled with Hoechst 33342 (Fig. 1a). We also confirmed that most Ki67-positive cells were localized in the same tumor regions (Fig. 1b). These data indicate that proliferating cancer cells with enough oxygen mainly express HIF-1α, even in tumors of orthotopically injected AsPC-1 cells.

We next examined whether HIF-1 is functional during normoxia in pancreatic cancer cells in vitro. Human pancreatic cancer AsPC-1, BxPC-3, and PANC-1 cells showed detectable HIF-1α protein levels during normoxia, as evidenced by western blotting (Fig. 1c). Knockdown (KD) of HIF-1α by small interfering RNA (siRNA) significantly diminished the reporter activity of hypoxia-responsive element-containing plasmids, which monitored HIF-1 activity in these cells during normoxia (Fig. 1d). Thus, HIF-1 was functional during normoxia in pancreatic cancer cells in vitro. FIH-1 suppresses the transcriptional activity of HIF-1 during normoxia, and Mint3 can inhibit FIH-1 in some cancer types [7, 11, 20]. Therefore, we further examined whether Mint3 is necessary for maintaining the transcriptional activity of HIF-1 during normoxia in pancreatic cancer cells. Mint3 depletion by short hairpin RNA (shRNA) did not affect HIF-1α and FIH-1 protein levels but decreased HIF-1 activity during normoxia (Fig. 1e–g). Mint3 depletion also decreased expression of the HIF-1 target genes *VEGFA* and *PDK1* (Supplementary Fig. S1). These results indicate that Mint3 is necessary for maintaining HIF-1 transcriptional activity during normoxia independently from HIF-1α protein levels in pancreatic cancer cells.

Pancreatic cancer cells were found to proliferate in regions with sufficient oxygen (Fig. 1b); thus, we examined whether Mint3 depletion affects their proliferation during normoxia. Interestingly, Mint3 depletion significantly decreased proliferation during normoxia in AsPC-1, BxPC-3, and PANC-1 cells (Fig. 1h). As decreased cell proliferation can be attributed to increased cell death and/or delayed cell cycle, we checked the expression levels of apoptosis-related proteins, but Mint3 depletion did not affect their expression (Supplementary Fig. S2a, b). Subsequently, we examined the cell cycle phase distribution of control and Mint3-depleted pancreatic cancer cells by propidium iodide staining and found that Mint3 depletion increased the G0/G1 population in AsPC-1 and BxPC-3 cells (Fig. 1i and Supplementary Fig. S2c, d). Thus, decreased proliferation of Mint3-depleted pancreatic cancer cells can be attributed to a delayed cell cycle.

### Mint3 regulates p21 and p27 protein levels in pancreatic cancer cells

**Fig. 2:**
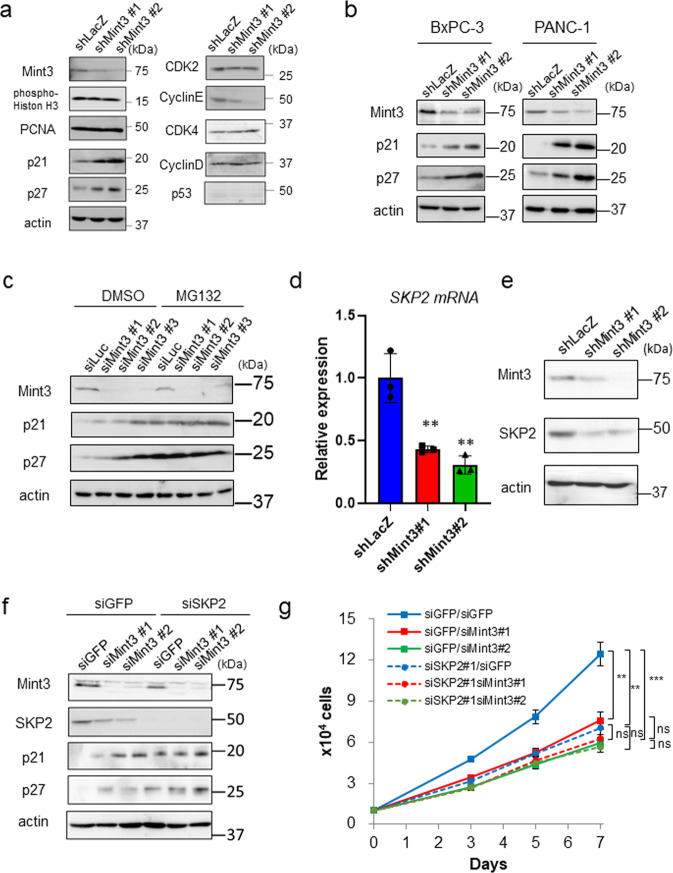
Mint3 knockdown increases p21 and p27 protein expression. **a** Immunoblotting of cell cycle-related proteins in control (shLacZ) and Mint3-depleted (shMint3) AsPC-1 cells. **b** p21 and p27 expression in control and Mint3-depleted BxPC-3 and PANC-1 cells. **c** p21 and p27 expression in AsPC-1 cells transfected with control siRNA (siLuc) or Mint3 siRNA (siMint3). Cells were treated with DMSO or MG132 (10 µM) for 4 h before lysis. **d** *SKP2* mRNA levels in control (shLacZ) and Mint3-depleted (shMint3) AsPC-1 cells. **e** Mint3, SKP2, and actin expression in shLacZ and shMint3 AsPC-1 cells. Mint3, SKP2, p21, p27, and actin expression (**f**) and cell growth (**g**) of Mint3 (siMint3)- and SKP2 (siSKP2)- depleted AsPC-1 cells. Error bars indicate SD (*n* = 3). **p < 0.01, ***p < 0.001, n.s., not significant (*t*-test).

We next examined the expression of cell cycle-related proteins in control and Mint3-depleted AsPC-1 cells. Among the tested proteins, p21 and p27 protein levels were found increased in Mint3-depleted AsPC-1, BxPC-3, and PANC-1 cells (Fig. 2a, b). p21 and p27 expression levels are commonly regulated via transcription and protein degradation [21, 22]. Given that Mint3-KD AsPC-1 and BxPC-3 cells showed comparable levels of p21 and p27 mRNA (Supplementary Fig. S3a, b), we analyzed their protein levels in the presence of the proteasomal inhibitor MG132. Transient depletion of Mint3 by siRNAs increased p21 and p27 protein levels in AsPC-1 and BxPC-3 cells compared with control siRNA (siLuc)-transfected cells (Fig. 2c and Supplementary Fig. S3c; DMSO). MG132 treatment further increased p21 and p27 protein levels and the difference in the protein levels between control and Mint3-depleted cells was negligible (Fig. 2c and Supplementary Fig. S3c; MG132). In addition, K48-linked ubiquitination levels of p21 and p27 proteins were decreased in Mint3-depleted AsPC-1 cells compared with control cells (Supplementary Fig. S3d). These results indicate that Mint3 regulates proteasomal degradation of p21 and p27 proteins in pancreatic cancer cells and that the Mint3 depletion-induced increase in p21 and p27 expression mediates cell cycle arrest, decreasing cell proliferation.

### Mint3 increases mRNA levels of ubiquitin ligase SKP2 in pancreatic cancer cells

SKP2 is the substrate recognition factor of the SCF ubiquitin ligase complex and degrades both p21 and p27 [23–27]. Interestingly, SKP2 mRNA and protein levels were decreased in Mint3-depleted pancreatic cancer cells (Fig. 2d, e and Supplementary Fig. S4a, b). Because SKP2 expression levels oscillate during the cell cycle [24, 28], decreased SKP2 may result from the altered distribution of cell cycle phases in Mint3-depleted pancreatic cancer cells. However, Mint3-KD AsPC-1 cells still showed decreased SKP2 mRNA levels even when cells were synchronized at the M phase by nocodazole treatment (Supplementary Fig. S4c, d). Thus, Mint3 can promote SKP2 expression independently of the cell cycle.

Next, we examined whether the increased p21/p27 levels and attenuated cell proliferation induced by Mint3 depletion depend on SKP2 in pancreatic cancer cells. SKP2 depletion by siRNAs increased p21 and p27 proteins and attenuated cell proliferation, but further Mint3 depletion did not affect these parameters in AsPC-1 cells (Fig. 2f, g). Although Mint3 depletion attenuates SKP2 expression in pancreatic cancer cells, whether this regulation is specific to cancer cells remains unclear. To address this, we analyzed the immortalized human pancreatic duct epithelial cell line H6c7. H6c7 cells expressed Mint3 at a comparable level to pancreatic cancer cells; however, Mint3 depletion did not affect SKP2 expression in H6c7 cells (Supplementary Fig. S5a, b). Thus, Mint3-mediated SKP2 expression is specific to pancreatic cancer cells. Taken together, the results indicate that Mint3 promotes SKP2 expression, thereby decreasing p21/p27 protein levels and enhancing cell proliferation in pancreatic cancer cells during normoxia.

### The Mint3-FIH-1 axis is involved in p21 and p27 regulation

**Fig. 3:**
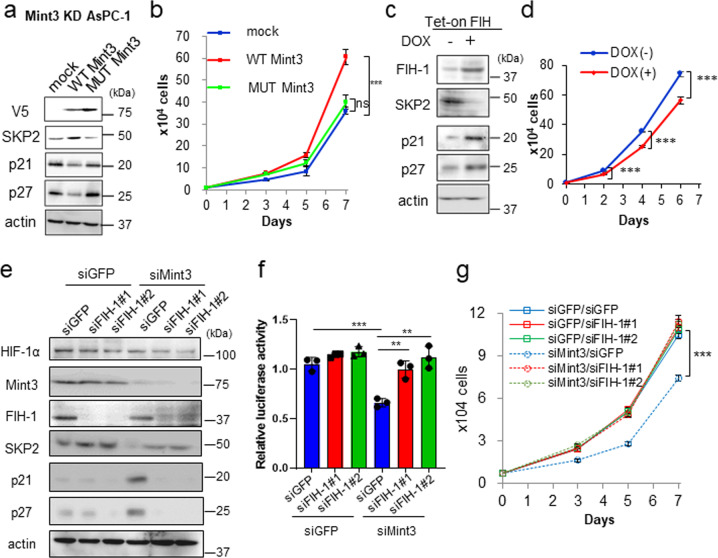
Mint3-FIH axis is involved in pancreatic cancer cell growth. SKP2, p21, and p27 expression (**a**) and cell growth (**b**) of Mint3 knockdown (KD) AsPC-1 cells expressing mock, V5-tagged WT, or MUT Mint3. SKP2, p21, and p27 expression (**c**) and cell growth of (**d**) doxycycline (DOX)-induced FIH-1 expressing AsPC-1 cells. AsPC-1 cells were treated with control siRNA (siGFP), Mint3 siRNA (siMint3), or FIH-1 siRNA (siFIH-1#1, #2), followed by immunoblotting (**e**), HIF-1 activity analysis (**f**), and cell growth assays (**g**). Error bars indicate SD (*n* = 3). **p < 0.01, ***p < 0.001, n.s., not significant (*t*-test).

Mint3 can activate HIF-1 by binding to and suppressing FIH-1 [11]. We confirmed that Mint3 binds to FIH-1 in pancreatic cancer cells by immunoprecipitation assays (Supplementary Fig. S6). Mint3 depletion attenuated HIF-1 activity in pancreatic cancer cells (Fig. 1g), but whether Mint3-mediated SKP2 expression and enhanced cell proliferation rely on the Mint3-FIH-1-HIF-1 axis remained unclear. To clarify this, we first focused on the relationship between Mint3 and FIH-1 by expressing V5-tagged wild-type (WT) Mint3 and its mutant (MUT), which has a markedly decreased binding ability to FIH-1 [15], in Mint3-KD AsPC-1, BxPC-3, and PANC-1 cells. Expression of WT Mint3, but not MUT Mint3, increased SKP2, decreased p21 and p27 protein levels, and restored cell proliferation in Mint3-KD pancreatic cancer cells (Fig. 3a, b and Supplementary Fig. S7a, b). Subsequently, whether overwhelming Mint3 by FIH-1 overexpression affects SKP2 expression and cell proliferation in pancreatic cancer cells was examined using the Tet-on expression system. Doxycycline-induced FIH-1 decreased SKP2, increased p21 and p27 levels, and attenuated cell proliferation (Fig. 3c, d and Supplementary Fig. S7c, d), similar to Mint3 depletion. Furthermore, FIH-1 KD reversed the decrease in HIF-1 activity and SKP2 expression, increase in p21 and p27 levels, and attenuation of cell proliferation induced by Mint3 depletion without affecting HIF-1α protein levels (Fig. 3e–g and Supplementary Fig. S7e–g). Altogether, these findings indicate that Mint3-mediated SKP2 expression and enhanced cell proliferation in pancreatic cancer cells are dependent on FIH-1 activity.

### Mint3 increases SKP2 mRNA levels in a HIF-1-dependent manner

**Fig. 4:**
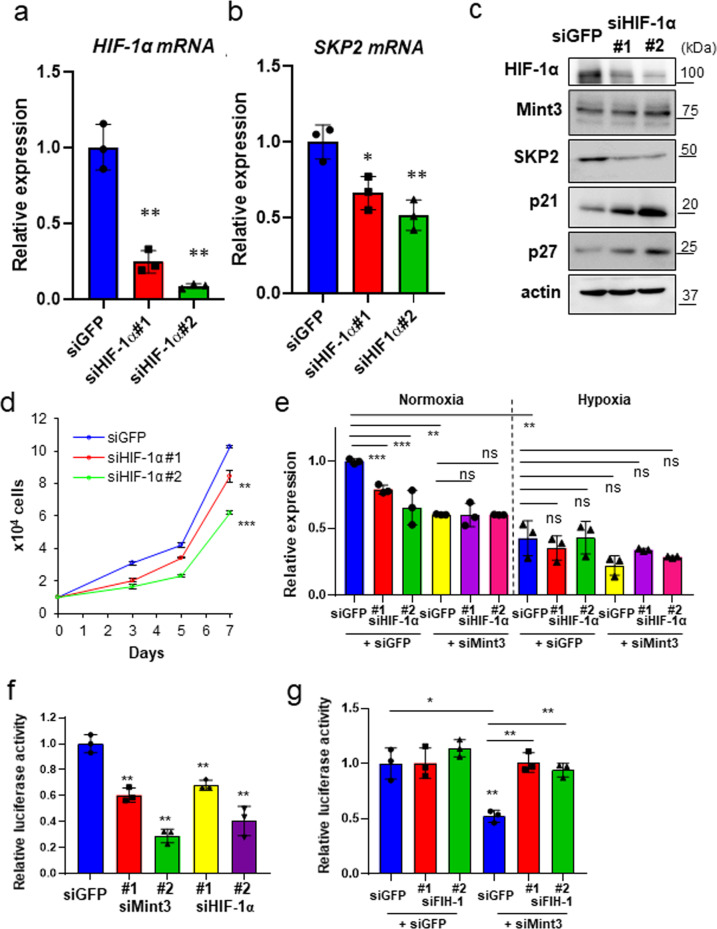
HIF-1α knockdown decreases SKP2 levels and pancreatic cancer cell growth. *HIF-1α* (**a**) and *SKP2* (**b**) mRNA levels in AsPC-1 cells transfected with control siRNA (siGFP) or HIF-1α siRNA (siHIF-1α #1, #2). **c** HIF-1α, SKP2, p21, and p27 expression in control (siGFP) and HIF-1α-depleted (siHIF-1α) AsPC-1 cells. **d** Cell growth of control (siGFP) and HIF-1α-depleted AsPC-1 cells. **e** mRNA levels of *SKP2* in AsPC-1 cells transfected with siGFP, siMint3, and siHIF-1α. **f** SKP2 promoter activity in AsPC-1 cells transfected with siGFP, siMint3, and siHIF-1α. **g** SKP2 promoter activity in AsPC-1 cells transfected with siGFP, siMint3, and siFIH-1. Error bars indicate SD (*n* = 3). *p < 0.05, **p < 0.01, ***p < 0.001, n.s., not significant (*t*-test).

As Mint3 controlled SKP2 mRNA levels in pancreatic cancer cells via FIH-1, we further examined whether Mint3-mediated SKP2 expression depends on HIF-1. HIF-1α depletion by siRNAs significantly decreased SKP2 mRNA levels in AsPC-1, BxPC-3, and PANC-1 cells (Fig. 4a, b and Supplementary Fig. S8a, b). HIF-1α depletion also increased p21 and p27 levels and attenuated cell proliferation in the pancreatic cancer cells (Fig. 4c, d and Supplementary Fig. S8c), similar to Mint3 depletion. Under depleted HIF-1α conditions, Mint3 depletion did not further decrease SKP2 mRNA levels in AsPC-1 cells (Fig. 4e; normoxia and Supplementary Fig. S8d). Interestingly, SKP2 mRNA levels in AsPC-1 cells decreased during hypoxia independently from HIF-1α and were not affected by Mint3 depletion (Fig. 4e; hypoxia). Thus, Mint3 increases SKP2 mRNA levels in a HIF-1-dependent manner specifically during normoxia. We further examined whether Mint3 and HIF-1α affect the promoter activity of SKP2 using a reporter assay and found that Mint3 or HIF-1α KD decreased SKP2 promoter activity in AsPC-1, BxPC-3, and PANC-1 cells; the Mint3-KD induced decrease in promoter activity was reversed by an additional KD of FIH-1 (Fig. 4f, g and Supplementary Fig. S8e, f). Thus, the Mint3/FIH-1/HIF-1α axis increases SKP2 mRNA levels, at least in part, by enhancing the promoter activity of SKP2 in pancreatic cancer cells.

### Mint3 depletion attenuates stemness features and chemoresistance in pancreatic cancer cells

**Fig. 5:**
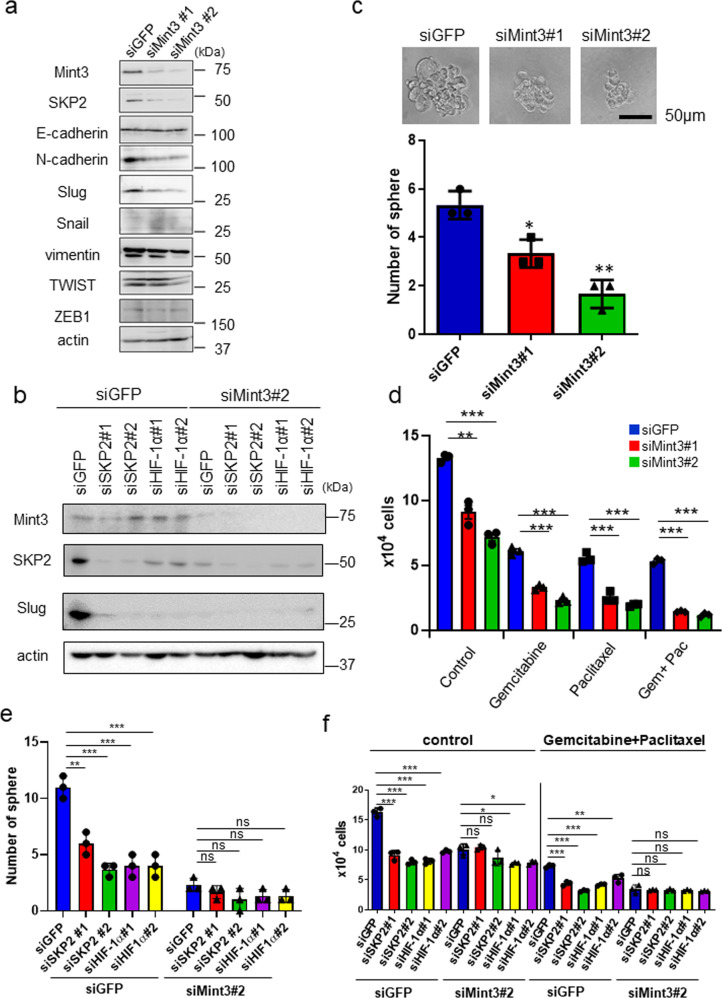
Mint3 depletion attenuates mesenchymal features, stemness, and chemoresistance in pancreatic cancer cells. **a** Immunoblotting of EMT-related proteins in control (siGFP) and Mint3-depleted (siMint3) AsPC-1 cells. **b** Slug expression in AsPC-1 cells transfected with siRNAs against GFP (siGFP), Mint3 (siMint3), SKP2 (siSKP2), and HIF-1α (siHIF-1α). **c** Sphere formation assay of control (siGFP) and Mint3-depleted (siMint3) AsPC-1 cells. Representative photos of tumor spheres (top). The number of spheres formed by control and Mint3-depleted AsPC-1 cells (bottom). **d** Control (siGFP) and Mint3-depleted (siMint3) AsPC-1 cells were counted after treatment with DMSO, gemcitabine (50 µM), paclitaxel (50 µM), or both gemcitabine and paclitaxel for 3 days. Sphere formation (**e**) and chemoresistance (**f**) of control (siGFP) and Mint3-depleted (siMint3) AsPC-1 cells transfected with control (siGFP), SKP2 (siSKP2), or HIF-1α (siHIF-1α) siRNA. Error bars indicate SD (*n* = 3). *p < 0.05, **p < 0.01, ***p < 0.001, n.s., not significant (*t*-test).

In addition to cell cycle regulation via p21 and p27, SKP2 is known to promote epithelial–mesenchymal transition (EMT), EMT-related stemness properties, and drug resistance in cancer cells by ubiquitinating various substrates [29–36]. HIF-1 also promotes EMT and stemness in cancer cells [37, 38]. Thus, we further examined whether Mint3 also controls EMT-related phenotypes in pancreatic cancer cells. At the mRNA level, Mint3 depletion increased expression of the epithelial marker E-cadherin and decreased expression of the mesenchymal markers N-cadherin, vimentin, and ZEB1 in AsPC-1 cells (Supplementary Fig. S9a). N-cadherin and vimentin mRNA levels were also decreased in Mint3-depleted BxPC-3 and PANC-1 cells (Supplementary Fig. S9b, c). At the protein level, Mint3 depletion decreased expression of several mesenchymal markers such as Slug, N-cadherin, and vimentin but did not affect E-cadherin levels in AsPC-1 cells (Fig. 5a). Although the effects of Mint3 depletion on N-cadherin and vimentin varied among cell lines, a consistent reduction in Slug protein levels was observed in Mint3-depleted BxPC-3 and PANC-1 cells, whereas Slug mRNA levels were maintained (Supplementary Fig. S9a–d). Moreover, Mint3 depletion attenuated pancreatic cancer cell migration (Supplementary Fig. S9e). Expression of Mint3 in Mint3-depleted AsPC-1 cells restored Slug expression in a dose-dependent manner (Supplementary Fig. S9f). Depletion of SKP2 and HIF-1α also decreased Slug levels (Fig. 5b), and SKP2 overexpression or FIH-1 KD reversed the decreased expression of Slug, N-cadherin, and vimentin in AsPC-1 cells (Supplementary Fig. S9g, h). These results indicate that Mint3 partially promotes EMT via the HIF-1/SKP2 pathway and that Slug may be a major target of Mint3-mediated EMT in pancreatic cancer cells.

EMT contributes to cancer stemness and chemoresistance [39, 40]. Mint3 depletion decreased tumor sphere formation ability and expression of the stemness-related genes SOX2, NANOG, and LGR5 in pancreatic cancer cells (Fig. 5c and Supplementary Fig. S10a–d). SKP2 overexpression or FIH-1 KD reversed this decreased expression of stemness-related genes in AsPC-1 cells (Supplementary Fig. S10e, f), indicating that the Mint3/HIF-1/SKP2 pathway promotes stemness-related gene expression. Mint3 depletion also sensitized pancreatic cancer cells to paclitaxel and gemcitabine, which are standard drugs used for pancreatic cancer treatment [41] (Fig. 5d and Supplementary Fig. S11a, b). Depletion of SKP2 and HIF-1α attenuated Mint3-mediated tumor sphere formation ability and chemoresistance in AsPC-1 cells (Fig. 5e, f). Thus, the Mint3-HIF-1-SKP2 axis is involved not only in the cell cycle but also in the stemness and chemoresistance of pancreatic cancer cells.

### Mint3 depletion in pancreatic cancer attenuates tumorigenicity in vivo

**Fig. 6:**
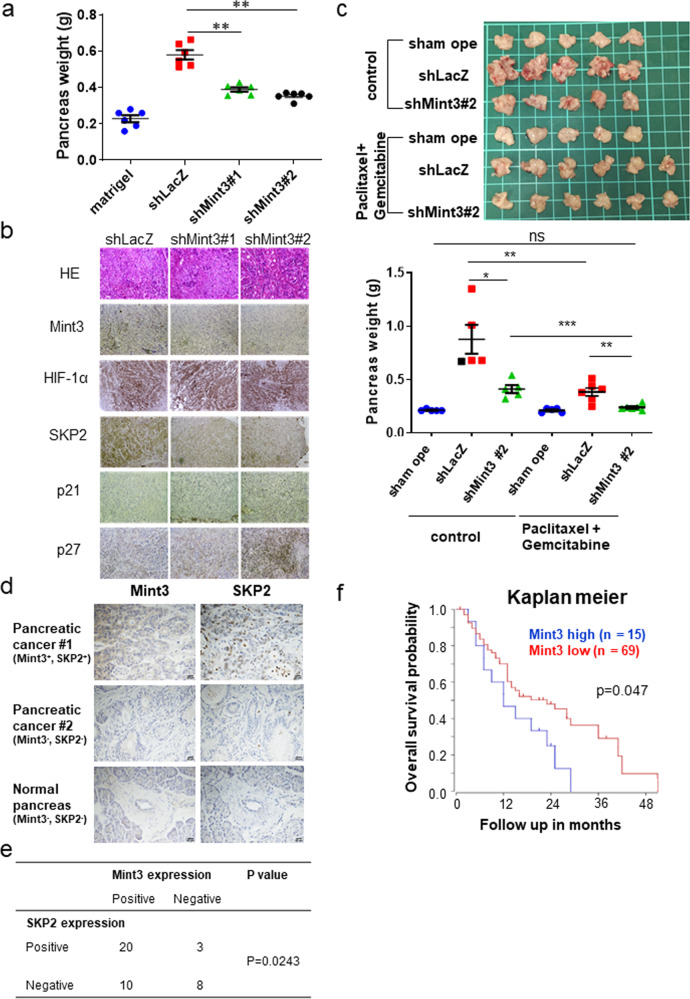
Mint3-depletion attenuates tumorigenicity of pancreatic cancer. **a** Tumor growth of orthotopically inoculated control (shLacZ) and Mint3-depleted (shMint3) AsPC-1 cells in the pancreas of immunodeficient mice (*n* = 6 for AsPC-1-inoculated groups and *n* = 5 for sham operation group). The data were analyzed by a Mann–Whitney *U* test. **b** H&E staining and immunostaining in pancreatic tumors of control and Mint3-depleted AsPC-1 cells. **c** Mice orthotopically inoculated with control and Mint3-depleted AsPC-1 cells were treated with paclitaxel (2.5 mg/kg b.w.) and gemcitabine (25 mg/kg b.w.) twice a week for 4 weeks. Photos of pancreas isolated from mice after chemotherapy (top). The weight of tumor-bearing pancreata was analyzed (bottom). *n* = 5–6 per group. Immunostaining of Mint3 and SKP2 in pancreatic cancer specimens. **d** Representative photos of immunostaining. **e** Correlation between Mint3 and SKP2 protein expression was statistically analyzed by the Chi-squared test. **f** Prognostic analysis of pancreatic cancer patients with high (*n* = 15, blue) or low (*n* = 69, red) Mint3 expression from a public dataset (Zhang 90 dataset) using the R2 database. *p* = 0.047 (log-rank test). Error bars indicate SD. **p < 0.01, ***p < 0.001, n.s. not significant.

Finally, we examined whether Mint3 contributes to the tumorigenicity of pancreatic cancer in vivo. Control (shLacZ) and Mint3-depleted (shMint3#1, #2) AsPC-1 cells were orthotopically injected into immunodeficient mice, after which tumor weights were analyzed 4 weeks after tumor inoculation. Weight of the tumor-bearing pancreas was significantly lower in the inoculated Mint3-depleted AsPC-1 cell group than in the control group (Fig. 6a). Moreover, FIH-1 depletion restored the tumorigenicity of Mint3-depleted AsPC-1 cells (Supplementary Fig. S12a). Mint3-depleted AsPC-1 tumors also showed reduced SKP2 levels and increased p21 and p27 levels but maintained HIF-1α, as evidenced by immunostaining (Fig. 6b). We further examined the expression levels of EMT-related genes in Mint3-depleted tumors of AsPC-1 cells and found increased E-cadherin mRNA levels and decreased N-cadherin and vimentin levels compared with those of control cells. Immunostaining further showed that N-cadherin, vimentin, and Slug expression decreased in tumors of Mint3-depleted AsPC-1 cells (Supplementary Fig. S12b, c).

Next, mice orthotopically inoculated with control or Mint3-depleted AsPC-1 cells were subjected to paclitaxel and gemcitabine chemotherapy for 4 weeks. Chemotherapy further decreased the weight of pancreata inoculated with Mint3-depleted AsPC-1 cells to similar levels of sham-operated pancreata (Fig. 6c), highlighting the efficacious combination of Mint3 depletion and chemotherapy for pancreatic cancer treatment. We further analyzed Mint3 expression in public clinical datasets and tissue microarrays of pancreatic cancer and found that Mint3 expression is positively correlated with SKP2 expression at mRNA and protein levels (Fig. 6d, e and Supplementary Fig. S12d). In addition, higher Mint3 expression was correlated with poorer prognosis of patients with pancreatic cancer in two independent datasets (Fig. 6f and Supplementary Fig. S12e). Altogether, the results indicate that Mint3 contributes to the tumorigenicity of pancreatic cancer in vivo.

## Discussion

**Fig. 7:**
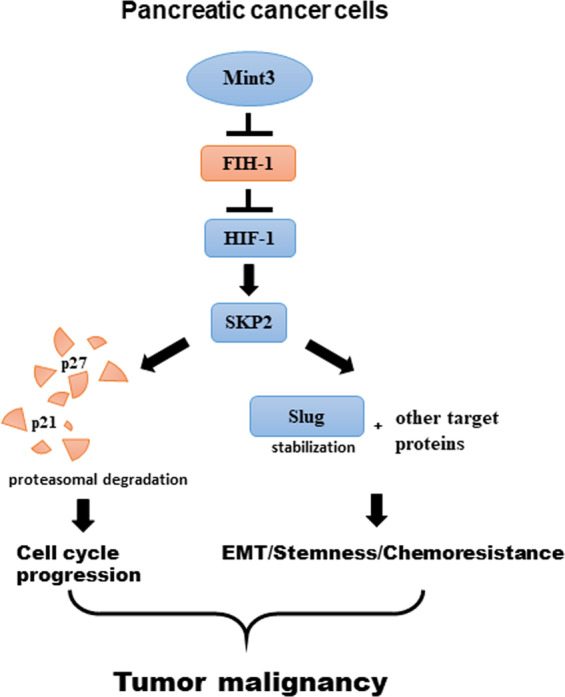
Schematic summary of malignant feature regulation by the Mint3-HIF-1-SKP2 axis in pancreatic cancer. Mint3 activates HIF-1, promoting SKP2 expression in pancreatic cancer cells during normoxia. Increased SKP2 levels promote cell proliferation by degrading p21 and p27, mesenchymal phenotypes, and EMT-related stemness/chemoresistance via other SKP2 targets. These malignant features contribute to tumor progression of pancreatic cancer.

Although the functions of HIF-1 during severe hypoxia have been extensively studied, its activity in the presence of sufficient oxygen remained unknown. Given that HIF-1 protein is mainly accumulated mainly in non-severe hypoxic areas of pancreatic cancer xenograft tumors, we were prompted to study how HIF-1 activity is maintained in the presence of oxygen and whether it contributes to cancer malignancy in pancreatic cancer. Here, we showed that HIF-1 activity during normoxia is maintained by Mint3 and that Mint3 depletion attenuates SKP2 expression via HIF-1 in pancreatic cancer cells. Mint3 depletion further affects tumor malignancy by attenuating cell proliferation, partial EMT, and chemoresistance in pancreatic cancer cells in vitro via decreased SKP2 expression. Mint3 depletion also attenuates tumorigenicity of human pancreatic cancer AsPC-1 cells. Thus, our study partly elucidated the biological importance of HIF-1 activity during normoxia, which is maintained by Mint3 in pancreatic cancer (Fig. 7). Nevertheless, we cannot exclude the possibility that HIF-1 target genes other than SKP2 also contribute to the Mint3-dependent tumorigenicity of pancreatic cancer.

Mint3 depletion attenuates SKP2 expression via HIF-1 in pancreatic cancer cells. However, whether this regulation is specific to cancer cells remains unclear. To address this, we examined human pancreatic duct epithelial H6c7 cells. Interestingly, although H6c7 cells expressed Mint3 at a comparable level to pancreatic cancer cells, Mint3 depletion did not affect SKP2 expression in H6c7 cells (Supplementary Fig. S5a, b). Mint3 requires the membrane-type matrix metalloprotease MT1-MMP to activate HIF-1, and MT1-MMP is highly expressed in malignant cancer [7]. Indeed, MT1-MMP expression was lower in H6c7 cells than in pancreatic cancer cells (Supplementary Fig. S5a). Thus, Mint3 may not be functional in normal pancreatic epithelial cells due to insufficient expression of Mint3 activators, such as MT1-MMP.

Interestingly, tissue microarray analyses of pancreatic tumor clinical specimens showed that Mint3 expression is also correlated with HIF-1α expression (Supplementary Fig. S13a, b). In pancreatic cancer cells, Mint3 depletion did not affect HIF-1α protein levels, and HIF-1α depletion also did not affect Mint3 expression. Inflammatory cytokines such as TNF-α can induce Mint3 expression in cancer-associated fibroblasts [42]. Moreover, NF-κB signaling promotes HIF-1α expression [43]. Thus, inflammation and/or other factors in the tumor microenvironment may affect both Mint3 and HIF-1α expression in pancreatic cancer, and co-overexpressed Mint3 and HIF-1α might further induce SKP2 overexpression and thereby promote pancreatic cancer progression.

SKP2 has oncogenic roles and is overexpressed in various types of cancers [18]. In pancreatic cancer, high SKP2 expression is correlated with poor prognosis [44], which is consistent with our database analysis of Mint3 expression in pancreatic cancer. Interestingly, our previous studies showed that Mint3 maintains HIF-1 activity during normoxia but does not affect in vitro cell proliferation of breast cancer MDA-MB-231 and fibrosarcoma HT1080 cells [14, 15, 17], which is in contrast to our findings in pancreatic cancer cells. In MDA-MB-231 and HT1080 cells, Mint3 did not affect SKP2 expression as well as p21/p27 levels and chemoresistance (Supplementary Fig. S14a–d), indicating the cell-type specific SKP2 regulation by the Mint3-HIF-1 axis. Further studies are needed to clarify whether and how genetic/epigenetic regulations allow Mint3/HIF-1 to induce SKP2 expression specifically in pancreatic cancer during carcinogenesis.

Among the tested EMT-related proteins, Mint3 depletion decreased Slug levels in pancreatic cancer cells in a HIF-1α- and SKP2-dependent manner. HIF-1 is known to activate Twist expression during hypoxia [37]. Our findings provide further evidence that HIF-1 contributes to EMT transcription factors even during normoxia in pancreatic cancer cells. Meanwhile, vimentin and N-cadherin levels were found partially decreased and other tested markers were not apparently affected in Mint3-depleted pancreatic cancer cells. Even though Mint3 partially contributes to EMT in pancreatic cancer cells, partial EMT is thought to be important as it provides flexibility of cell states, thereby promoting cancer malignancy [39, 40]. This may explain the attenuated stemness and chemoresistance in Mint3-depleted pancreatic cancer cells. The stability of Twist is promoted via its K63-linked ubiquitination by SKP2 in prostate cancer [35]. Interestingly, K63-linked ubiquitination levels of Slug were decreased and MG132 treatment restored the decreased Slug protein levels in Mint3-depleted AsPC-1 cells (Supplementary Fig. S15), indicating that Mint3 promotes the stability of Slug proteins. Although Twist protein was not affected by Mint3 depletion in AsPC-1 cells, Mint3-mediated SKP2 expression may control the stability of Slug protein in the same fashion as Twist protein in prostate cancer.

It is known that proliferating cells are susceptible to chemotherapy. However, Mint3-depleted pancreatic cancer cells show both attenuated cell proliferation and increased susceptibility to paclitaxel and gemcitabine chemotherapy compared with that of control cells. Because the G0/G1 arrest induced by Mint3 depletion was partial, attenuation of EMT-mediated chemoresistance may overcome the effect of delayed cell proliferation on the susceptibility to chemotherapy in Mint3-depleted pancreatic cancer cells.

In conclusion, we showed that Mint3 depletion in cancer cells hampers cell proliferation, EMT, cancer stemness, and chemoresistance via the HIF-1-SKP2 axis during normoxia in pancreatic cancer cells. Moreover, Mint3-deficient mice show no apparent abnormalities or decreased macrophage-mediated metastasis [12, 13, 45]. Thus, our findings altogether indicate that Mint3 is a possible target for pancreatic cancer therapy without severe adverse effects.

## Materials and methods

Detailed information on Material and methods are available in Supplementary Information and Supplementary Table S1–S6.

## Supplementary information

Supplementary Information

Supplementary Figure 1

Supplementary Figure 2

Supplementary Figure 3

Supplementary Figure 4

Supplementary Figure 5

Supplementary Figure 6

Supplementary Figure 7

Supplementary Figure 8

Supplementary Figure 9

Supplementary Figure 10

Supplementary Figure 11

Supplementary Figure 12

Supplementary Figure 13

Supplementary Figure 14

Supplementary Figure 15

Supplementary Table 1

Supplementary Table 2

Supplementary Table 3

Supplementary Table 4

Supplementary Table 5

Supplementary Table 6
