# Supplementary material for: Mint3 depletion restricts tumor malignancy of pancreatic cancer cells by decreasing SKP2 expression via HIF-1
Source: Oncogene. 2020 Aug 21;39(39):6218–30. doi: 10.1038/s41388-020-01423-8 (PMC7515798; doi:10.1038/s41388-020-01423-8)
Supplement: Supplementary file 1 — Supplementary Information [file 41388_2020_1423_MOESM1_ESM.docx]

**Supplementary Information**

**Mint3 depletion restricts tumor malignancy of pancreatic cancer cells by decreasing SKP2 expression via HIF-1**

**Akane Kanamori^1^, Daisuke Matsubara^2^, Yurika Saitoh^1,3^, Yuya Fukui^1^, Noriko Gotoh^4^, Shuichi Kaneko^5^, Motoharu Seiki^6^, Yoshinori Murakami^7^, Jun-ichiro Inoue^1^, Takeharu Sakamoto^8*^**

^1^Division of Cellular and Molecular Biology, the Institute of Medical Science, The University of Tokyo, Shirokanedai, Minato-ku, Tokyo, Japan

^2^ Departments of Pathology, Jichi Medical University, Yakushiji, Shimotsuke-shi, Tochigi, Japan.

^3^Center for Medical Education, Teikyo University of Science, Senjusakuragi, Adachi-ku, Tokyo, Japan

^4^Division of Cancer Cell Biology, Cancer Research Institute, Kanazawa University, Kakuma-machi, Kanazawa, Ishikawa, Japan

^5^Department of Gastroenterology, Institute of Medical, Pharmaceutical and Health Sciences, Kanazawa University, Takaramachi, Kanazawa, Ishikawa, Japan

^6^Division of Cancer Cell Research, the Institute of Medical Science, The University of Tokyo, Shirokanedai, Minato-ku, Tokyo, Japan

^7^Division of Molecular Pathology, the Institute of Medical Science, The University of Tokyo, Shirokanedai, Minato-ku, Tokyo, Japan

^8^Department of System Biology, Institute of Medical, Pharmaceutical and Health Sciences, Kanazawa University, Takaramachi, Kanazawa, Ishikawa, Japan

**^*^Address correspondence to:** Takeharu Sakamoto, Ph. D., 13-1 Takara-machi, Kanazawa, Ishikawa 920-8640, Japan. Tel: +81-76-265-2235; Fax: +81-76-234-4250; E-mail: t-saka@staff.kanazawa-u.ac.jp

**Supplementary Materials and Methods**

**Cell culture**

Human pancreas adenocarcinoma AsPC-1, BxPC-3, and PANC-1, breast cancer MDA-MB-231, and fibrosarcoma HT1080 cells were purchased from the America Type Culture Collection (Manassas, VA, USA). AsPC-1, BxPC-3, and PANC-1 were cultured in RPMI 1640 medium (Thermo Fisher Scientific, Waltham, MA, USA) supplemented with 10% FBS and penicillin-streptomycin (Thermo Fisher Scientific). MDA-MB-231, HT1080, and 293FT cells were cultured in Dulbecco’s modified Eagle’s medium (DMEM; Thermo Fisher Scientific) supplemented with 10% FBS and penicillin-streptomycin. The immortalized human pancreatic duct epithelial cell line H6c7 was purchased from Kerafast (Boston, MA, USA) and cultured in Keratinocyte SFM (Thermo Fisher Scientific, Waltham, MA, USA). All cell lines were routinely tested to exclude mycoplasma contamination. For experiments conducted under hypoxic conditions, cells were cultured with 1% O_2_ and 5% CO_2_ in a Model 9200 incubator (Wakenyaku, Kyoto, Japan) for 24 h as previously described [1].

**Reagents**

MG132 was purchased from Cell Signaling Technology (Danvers, MA, USA); gemcitabine was from Tokyo Chemical Industry (Tokyo, Japan); and nocodazole and staurosporine were obtained from Merck (Kenilworth, NJ, USA). B27 supplement, bFGF, EGF, and paclitaxel were purchased from FUJIFILM WAKO (Osaka, Japan).

**Cell growth assay**

Cells (0.25–1 × 10^4^) were seeded onto a 24-well plate and cultured at 37 °C in a humidified CO_2_ incubator. The cells were counted periodically using a hemocytometer.

**Cell cycle analysis**

Cells were seeded in 6-well plates at 1 × 10^5^ cells per well and cultured overnight. Cells were harvested and fixed with 70% ethanol at -20 °C overnight, stained with Tali cell cycle solution, and analyzed using the Tali Image Cytometer (Thermo Fisher Scientific).

**Tumor sphere formation assay**

AsPC-1 cells were seeded onto 96-well ultra-low attachment plates in DMEM/F-12 medium (Thermo Fischer Scientific) supplemented with 100 μg/mL EGF, 100 μg/mL bFGF, and 2% B27 supplement at a density of 250 cells per well. The appearance of tumor spheres was evaluated after 7 days. Tumor spheres with diameters of > 50 μm were manually counted under a microscope.

**In vivo tumorigenesis assay**

Experimental protocols were approved by the Animal Care and Use Committees of the Institute of Medical Science, University of Tokyo. Sample size is based on statistical analysis of variance and on exploratory experiments. Cell tumorigenicity was assessed using 8-week-old male BALB/c nude mice (CLEA Japan, Tokyo, Japan). Mice were anesthetized with butorphanol (Meiji Seika, Tokyo, Japan), medetomidine (Fujita Pharmaceutical company, Tokyo, Japan), and midazolam (Sandoz, Tokyo, Japan), after which a 1 cm long longitudinal incision was made into the left upper quadrant of the abdominal cavity. AsPC-1 (1 × 10^6^) cell suspension (25 µL) was mixed with the same volume of Matrigel (Corning Inc., Corning, NY, USA) and injected into the pancreatic parenchyma using an ice-cold 27-gauge needle. The pancreas was gently placed back into the abdominal cavity and the surgical opening was closed using wound clips (BD Biosciences, Franklin Lakes, NJ, USA). The weight of the tumor-bearing pancreas was measured 4 weeks after transplantation. For combination treatment of paclitaxel and gemcitabine, mice were intraperitoneally injected twice a week with paclitaxel (2.5 mg/kg body weight [b.w.]) and gemcitabine (25 mg/kg b.w.) or vehicle1 week after transplantation. Chemotherapy was performed for 4 weeks. All mice were included in the analysis. Investigators were not blinded during data acquisition and analysis.

**Plasmids**

shRNA sequences are listed in Supplementary Table S1. Targeted gene sequences were subcloned as deoxyribose fragments into pENTR/TOPO vector (Thermo Fisher Scientific) and recombined into lentivirus pLenti6 BLOCKiT vector (Thermo Fisher Scientific). shRNA-expressing lentiviral vectors were generated and used as previously described [2, 3]. Human wild-type Mint3 and mutant Mint3 expression vectors were prepared as previously described [1, 4, 5]. Human FIH-1 cDNA was obtained from MDA-MB-231 cells by reverse transcription (RT)-PCR using the following primer pairs, forward, 5ʹ-CCCTCGTAAAGAATTCATGGCGGCGACAGCG GCGGA-3ʹ and reverse, 5ʹ-GAGGTGGTCTGGATCCCTAGTTGTATCGGCCCTTGA-3ʹ. FIH-1 cDNA was subcloned into pGEM-T easy vector (Promega, Madison, WI, USA) using the BamHI and EcoRI restriction sites of pRetroX-TetOne vector (TaKaRa Bio, Shiga, Japan). The lentiviral expression vectors were generated and used according to manufacturer’s instructions. For induction experiments, cells were treated with 100 ng/mL doxycycline (TaKaRa Bio) for 48 h.

**Immunoprecipitation**

To detect ubiquitination, AsPC-1 cells were lysed with lysis buffer (1% Nonidet P-40, 50 mM Tris pH 8.0, and 150 mM NaCl) and centrifuged at 15,000 × *g* for 15 min at 4 °C. The supernatants were collected and incubated with rabbit anti-p21 polyclonal antibody (Cell Signaling Technology, Danvers, MA, USA), rabbit anti-p27 polyclonal antibody (Cell Signaling Technology), or rabbit anti-Slug polyclonal antibody (Cell Signaling Technology) overnight at 4 °C. Then, Protein G agarose (Thermo Fisher Scientific) was added to the lysate for 1 h at 4 °C. The beads were washed four times with lysis buffer, and then the proteins were eluted with sample buffer and analyzed via immunoblotting with K48-linkage Specific Polyubiquitin (D9D5) Rabbit mAb (#8081; Cell Signaling Technology) or K63-linkage Specific Polyubiquitin (D7A11) Rabbit mAb (#5621; Cell Signaling Technology). To detect interactions between Mint3 and FIH-1, immunoprecipitation was performed as previously described [4, 6] using rabbit anti-FIH-1 antibody (NB100-428, Novus Biologicals, Littleton, CO, USA).

**siRNA knockdown**

Knockdown by siRNA was carried out using Lipofectamine™ RNAiMAX (Thermo Fisher Scientific) as previously described [1]. Target sequences are listed in Supplementary Table 2.

**Western blotting**

Cell lysates were prepared and subjected to western blotting as previously described [2] using specific antibodies (Supplementary Table 3). Nuclear extracts were prepared using a Nuclear Extract Kit (Active Motif, Carlsbad, CA, USA).

**RNA isolation, reverse transcription, and real-time PCR**

Total RNA was isolated from cells using the RNeasy Mini Kit (Qiagen, Hilden, Germany) and reverse transcribed using Superscript III (Thermo Fisher Scientific) and random primers. The resulting cDNA was then analyzed by real-time PCR in a 7500 Real-Time PCR System (Thermo Fisher Scientific) using ReverTra Ace qPCR RT Master Mix (TOYOBO, Osaka, Japan); primers are listed in Supplementary Table 4. Expression levels were normalized to *ACTB*.

**Luciferase assay**

HIF-1 activity was measured using pGL4.42 vectors that express firefly luciferase under the control of a hypoxia response element (HRE; Promega). To generate the SKP2 promoter vector, the SKP2 promoter region (2000 bp upstream the SKP2 translation starting site) was subcloned into the pGEM-T easy vector (Promega) using the following primer pairs, forward, 5ʹ-GGTACCCAGGTAACTCCTAGTAAAACCAAGA-3ʹ and reverse, 5ʹ-CTCGAGAGCGTCCGCAGGCCCGGGAGTTTAA-3ʹ and the KpnI and XhoI restriction sites of the pGL3 luciferase reporter vector (Promega). A pRL vector expressing Renilla luciferase (Promega) served as an internal control. Cells (1 × 10^5^ per well) were seeded onto 24-well plates and co-transfected with a reporter plasmid (100 ng) and the internal control pRL vector (10 ng) using Lipofectamine 2000 (Thermo Fisher Scientific). The luciferase activity of transfected cell lysates was measured using the Dual Luciferase Reporter Assay System (Promega) according to manufacturer’s instructions. Luminescence was measured using the GloMax 20/20 Luminometer (Promega).

**Transwell migration assay**

Transwell migration assays were performed as described previously [7] with some modifications. Briefly, Transwells with 8 µm pore size filters (Corning Inc., Corning, NY, USA) were inserted into 24-well plates. RPMI 1640 medium (500 µL) containing 10% FBS was added to the lower chamber while a 200-µL cell suspension (5 × 10^4^ cells) was added to the upper chamber. The plates were incubated at 37 ºC in a 5% CO_2_ atmosphere for 4 h. Cells in the lower chamber were then stained with 0.1% crystal violet solution (Sigma-Aldrich, St. Louis, MO, USA) and counted.

**Evaluation of immunohistochemistry**

Evaluation of immunohistochemical staining via light microscopy was performed by a pathologist (D.M) blinded to the clinical data of each patient. Nuclear staining was assessed for SKP2, while cytoplasmic staining was assessed for Mint3. SKP2 immunoreactivity was evaluated as either high- or low-level expression, with a cut-off value of 5% positively stained cancer cells. Mint3 immunoreactivity was evaluated semi-quantitatively based on the intensity and estimated percentage of tumor cells that were stained. Intensity was quantified as follows: 1+, weak staining (detection required high magnification); 2+, moderate staining (detected readily at medium magnification); 3+, strong staining (detected readily at low magnification). The percentages of positive cells were scored into five categories: 0, 0%; 1, 1–25%; 2, 26–50%; 3, 51–75%; 4, 76–100%. The product of the intensity and percentage scores was used as the final staining score. The final score for Mint3 was defined as low-level expression (final staining score < 5) and high-level expression (final staining score ≥ 5).

**Hematoxylin and eosin (H&E) staining and immunohistochemistry of paraffin-embedded sections**

Tissue specimens from xenografted tumors were fixed in 4% PFA/PBS for 4 h, followed by dehydration in an increasing graded ethanol series and clearing with xylene. The specimens were subsequently embedded in paraffin wax in cassettes for tissue sectioning. Sections were cut at a thickness of 3 μm and subjected to standard H&E staining or immunostaining. For immunostaining, the sections were first treated with 0.3% hydrogen peroxide in PBS for 1 h to block endogenous peroxidase activity. After blocking with 5% normal goat serum/PBS, sections were incubated with the specific antibodies (Supplementary Table 5). The primary antibody was detected using the DAKO Envision Kit (Dako, Glostrup, Denmark) according to manufacturer’s instructions. All sections were counterstained with hematoxylin.

For tissue microarray analysis, immunohistochemical staining was carried out on a paraffin-embedded pancreatic cancer tissue array purchased from US BIOMAX (PA484 for Mint3 and SKP2 expression, PA484A for Mint3 and HIF-1α expression; Derwood, MD, USA). Sections were deparaffinized in xylene and rehydrated in a decreasing graded ethanol series. Antigen retrieval was carried out for SKP2 immunostaining by autoclaving in 10 mM citrate buffer (pH 6.0) for 10 min at 121 °C, followed by cooling for 20 min. After blocking endogenous peroxidase activity with a 3% aqueous H_2_O_2_ solution for 5 min, the sections were incubated with rabbit monoclonal anti-human SKP2 (1:100; #2652; Cell Signaling Technology) , mouse monoclonal anti-human Mint3 (1:100; #611380; BD Biosciences), and rabbit anti-HIF-1α antibody (1:100; #NB100-479; Novus Biologicals) antibodies at 4 °C overnight. After washing with TBS buffer, SKP2 and Mint3 antibodies were detected using the DAKO Envision+ Dual Link System. 3,3ʹ-Diaminobenzidine tetrahydrochloride (DAB) was used as a chromogen, while hematoxylin was used as a light counterstain.

**Immunostaining**

Mice were intravenously injected with pimonidazole (100 mg/kg b.w.; Hypoxyprobe, Burlington, MA, USA) and Hoechst 33342 (10 mg/kg b.w.; Merck, Kenilworth, NJ, USA) 90 min and 10 min before sacrifice, respectively. For histological analysis, frozen samples were cut into 10-μm thick sections and fixed in 4% PFA. Frozen sections of tumor tissues were prepared and subjected to immunostaining using specific antibodies (Supplementary Table 6) as previously described [2]. The sections were imaged using confocal microscopy (Nikon, Tokyo, Japan).

**Database analysis**

Prognostic and gene expression correlation analyses of human pancreatic cancer datasets were performed using R2: Genomics Analysis and Visualization Platform (http://r2.amc.nl). For prognostic analysis, Zhang 96 (GSE28735) [8] and Bailey 96 datasets [9] were analyzed. For correlation analysis of Mint3 and SKP2 mRNA expression, the Badea 78 dataset [10] was analyzed.

**Statistical analyses.**

Sample size is based on statistical analysis of variance and on exploratory experiments. All data are presented as mean ± s.d. Data were analyzed by using the two-sided unpaired *t* test with Welch’s correction, the Mann-Whitney U test, or the Chi-squared test using GraphPad Prism 7 software (GraphPad Software, Inc., La Jolla, CA, USA). p < 0.05 was considered as statistically significant.

**References**

1 Sakamoto T, Niiya D, Seiki M. Targeting the Warburg effect that arises in tumor cells expressing membrane type-1 matrix metalloproteinase. *J Biol Chem* 2011; 286: 14691-14704.

2 Yoshino S, Hara T, Nakaoka HJ, Kanamori A, Murakami Y, Seiki M *et al*. The ERK signaling target RNF126 regulates anoikis resistance in cancer cells by changing the mitochondrial metabolic flux. *Cell Discov* 2016; 2: 16019.

3 Yoshino S, Hara T, Weng JS, Takahashi Y, Seiki M, Sakamoto T. Genetic Screening of New Genes Responsible for Cellular Adaptation to Hypoxia Using a Genome-Wide shRNA Library. *PLoS ONE* 2012; 7: e35590.

4 Sakamoto T, Seiki M. Mint3 enhances the activity of hypoxia-inducible factor-1 (HIF-1) in macrophages by suppressing the activity of factor inhibiting HIF-1. *J Biol Chem* 2009; 284: 30350-30359.

5 Sakamoto T, Weng JS, Hara T, Yoshino S, Kozuka-Hata H, Oyama M *et al*. Hypoxia-inducible factor 1 regulation through cross talk between mTOR and MT1-MMP. *Mol Cell Biol* 2014; 34: 30-42.

6 Nakaoka HJ, Hara T, Yoshino S, Kanamori A, Matsui Y, Shimamura T *et al*. NECAB3 Promotes Activation of Hypoxia-inducible factor-1 during Normoxia and Enhances Tumourigenicity of Cancer Cells. *Sci Rep* 2016; 6: 22784.

7 Hara T, Mimura K, Abe T, Shioi G, Seiki M, Sakamoto T. Deletion of the Mint3/Apba3 gene in mice abrogates macrophage functions and increases resistance to lipopolysaccharide-induced septic shock. *J Biol Chem* 2011; 286: 32542-32551.

8 Zhang G, Schetter A, He P, Funamizu N, Gaedcke J, Ghadimi BM *et al*. DPEP1 inhibits tumor cell invasiveness, enhances chemosensitivity and predicts clinical outcome in pancreatic ductal adenocarcinoma. *PLoS One* 2012; 7: e31507.

9 Bailey P, Chang DK, Nones K, Johns AL, Patch AM, Gingras MC *et al*. Genomic analyses identify molecular subtypes of pancreatic cancer. *Nature* 2016; 531: 47-52.

10 Badea L, Herlea V, Dima SO, Dumitrascu T, Popescu I. Combined gene expression analysis of whole-tissue and microdissected pancreatic ductal adenocarcinoma identifies genes specifically overexpressed in tumor epithelia. *Hepatogastroenterology* 2008; 55: 2016-2027.
