# Supplementary material for: Mint3 depletion restricts tumor malignancy of pancreatic cancer cells by decreasing SKP2 expression via HIF-1
Source: Oncogene. 2020 Aug 21;39(39):6218–30. doi: 10.1038/s41388-020-01423-8 (PMC7515798; doi:10.1038/s41388-020-01423-8)
Supplement: Supplementary file 2 — Supplementary Figure 1 [file 41388_2020_1423_MOESM2_ESM.pdf]

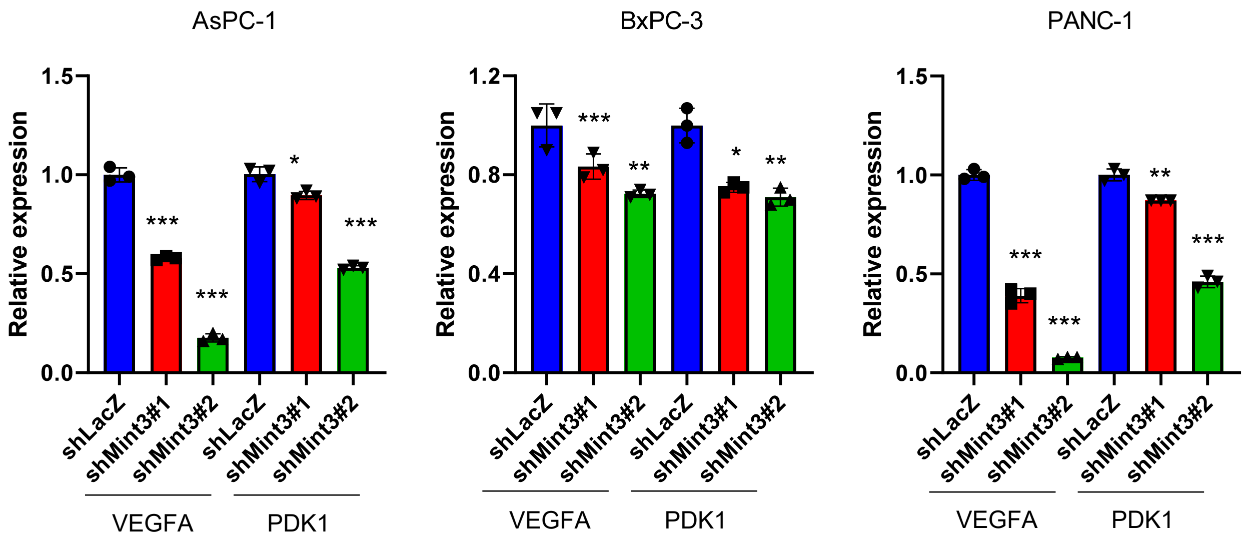

Supplementary Figure 1. Mint3 depletion attenuates expression of HIF-1 target genes in pancreatic cancer cells.
mRNA levels of the HIF-1 target genes *VEGFA* and *PDK1* in control (shLacZ) and Mint3-depleted (shMint3#1, #2) pancreatic cancer cells. Error bars indicate SD (n = 3). *$p < 0.05$, **$p < 0.01$, ***$p < 0.001$ (t-test).
