# Supplementary material for: Mint3 depletion restricts tumor malignancy of pancreatic cancer cells by decreasing SKP2 expression via HIF-1
Source: Oncogene. 2020 Aug 21;39(39):6218–30. doi: 10.1038/s41388-020-01423-8 (PMC7515798; doi:10.1038/s41388-020-01423-8)
Supplement: Supplementary file 3 — Supplementary Figure 2 [file 41388_2020_1423_MOESM3_ESM.pdf]

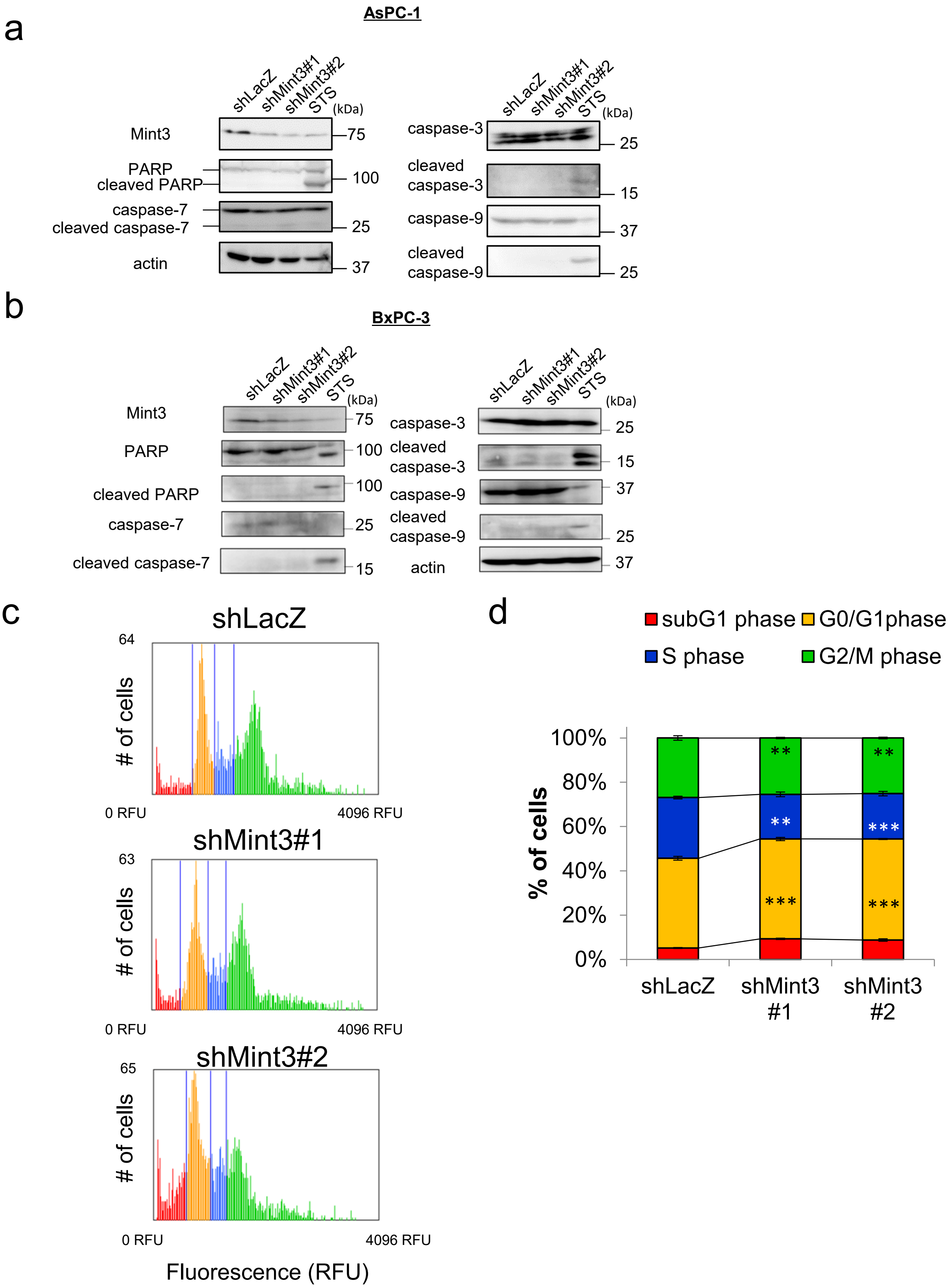

Supplementary Figure 2. Mint3 depletion does not affect expression of apoptosis-related proteins in pancreatic cancer cells.

(**a**, **b**) Immunoblotting of apoptosis-related proteins in control (shLacZ) and Mint3-depleted (shMint3) AsPC-1 (**a**) and BxPC-3 cells (**b**). Staurosporine (STS; 500 nM) treatment for 5 h was used as a positive control for apoptosis.

(**c**, **d**) Cell cycle analysis of control and Mint3-depleted BxPC-3 cells. (**c**) Representative graphs of propidium iodide staining. (**d**) The distribution of each cell cycle phase in BxPC-3 cells was analyzed. Error bars indicate SD (n = 3). * $p < 0.01$, *** $p < 0.001$ (*t*-test).
