# Supplementary material for: Mint3 depletion restricts tumor malignancy of pancreatic cancer cells by decreasing SKP2 expression via HIF-1
Source: Oncogene. 2020 Aug 21;39(39):6218–30. doi: 10.1038/s41388-020-01423-8 (PMC7515798; doi:10.1038/s41388-020-01423-8)
Supplement: Supplementary file 4 — Supplementary Figure 3 [file 41388_2020_1423_MOESM4_ESM.pdf]

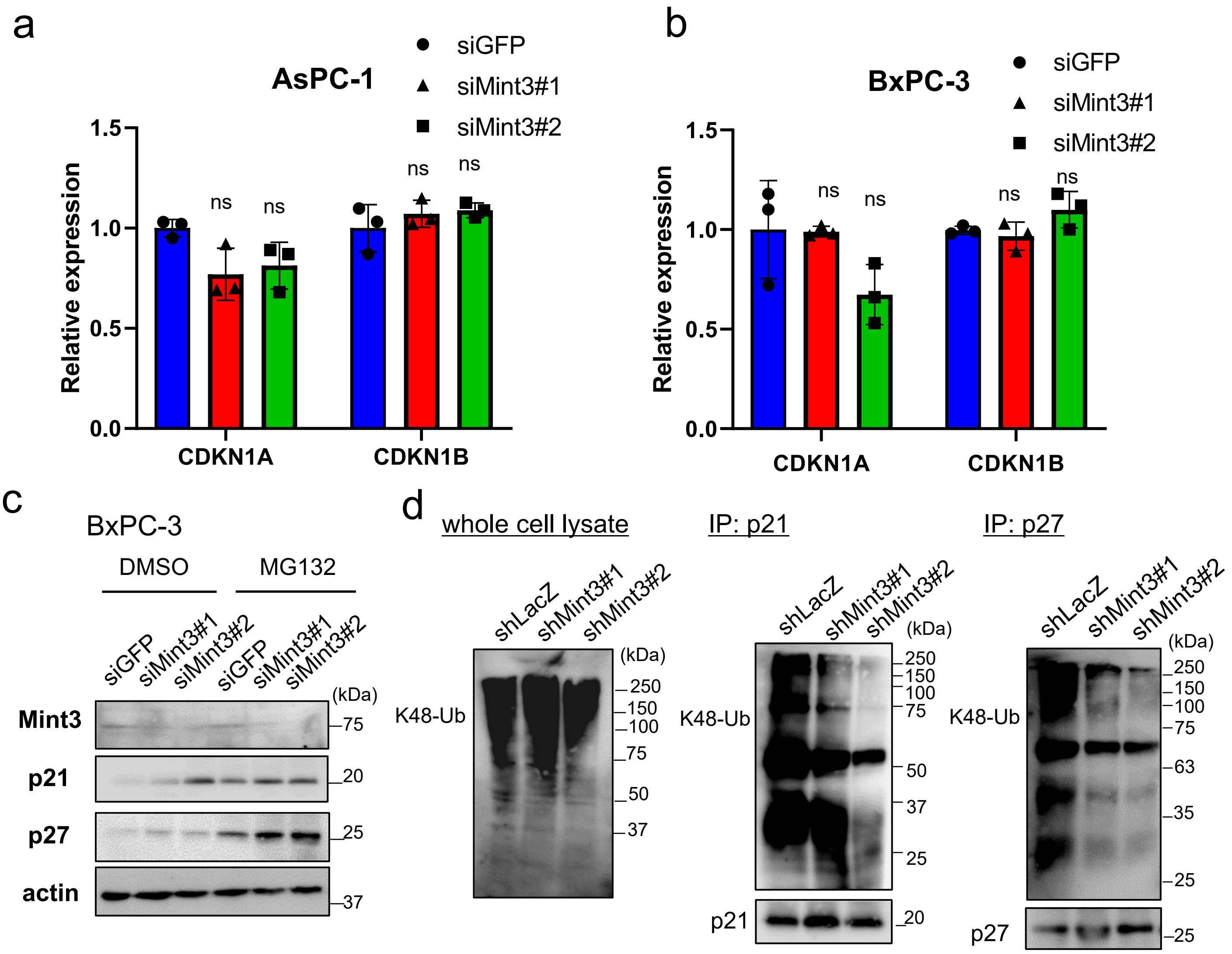

Supplementary Figure 3. Mint3 depletion increases p21 and p27 protein levels in pancreatic cancer cells.
(**a**, **b**) *CDKN1A* (encoding p21) and *CDKN1B* (encoding p27) mRNA levels in control and Mint3-depleted AsPC-1 (**a**) and BxPC-3 cells (**b**). Expression levels were normalized to *ACTB*. Error bars indicate SD (n = 3). ns, not significant (*t*-test).
(**c**) Immunoblotting of p21 and p27 in BxPC-3 cells transfected with control siRNA (siLuc) or Mint3 siRNA (siMint3). Cells were treated with DMSO or MG132 (10 mM) for 4 h before lysis.
(**d**) p21 and p27 proteins in control (shLacZ) and Mint3-depleted (shMint3#1, #2) AsPC-1 cells were immunoprecipitated and detected using K48-linkage specific polyubiquitin antibody. Note that K48-linkage polyubiquitination levels of p21 and p27 proteins were decreased in Mint3-depleted AsPC-1 cells.
