# Supplementary material for: Mint3 depletion restricts tumor malignancy of pancreatic cancer cells by decreasing SKP2 expression via HIF-1
Source: Oncogene. 2020 Aug 21;39(39):6218–30. doi: 10.1038/s41388-020-01423-8 (PMC7515798; doi:10.1038/s41388-020-01423-8)
Supplement: Supplementary file 5 — Supplementary Figure 4 [file 41388_2020_1423_MOESM5_ESM.pdf]

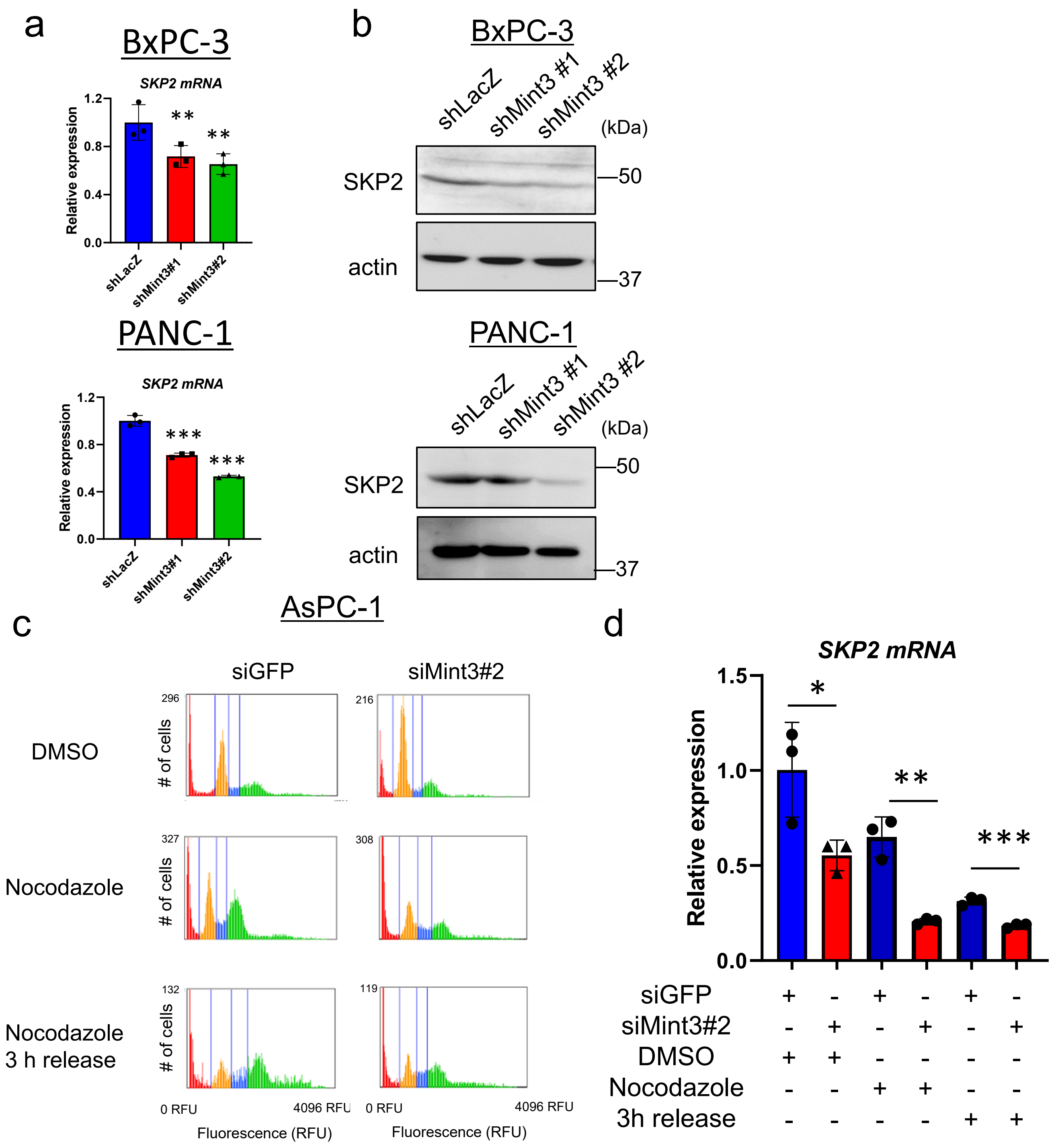

Supplementary Figure 4. Mint3 depletion decreases SKP2 mRNA levels in pancreatic cancer cells.
(**a**) *SKP2* mRNA levels in control (shLacZ) and Mint3-depleted (shMint3) BxPC-3 and PANC-1 cells. Expression levels were normalized to *ACTB*.
(**b**) SKP2 protein expression in control (shLacZ) and Mint3-depleted BxPC-3 and PANC-1 cells.
(**c**, **d**) Representative PI staining (**c**) and *SKP2* mRNA levels (**d**) in control (siGFP) and Mint3-depleted (siMint3) AsPC-1 cells were synchronized by nocodazole (10 μM) treatment for 16 h and cultured without nocodazole for 3 h after nocodazole treatment.
Error bars indicate SD (n = 3). *$p < 0.05$, **$p < 0.01$, ***$p < 0.001$ (*t*-test).
