# Supplementary material for: Mint3 depletion restricts tumor malignancy of pancreatic cancer cells by decreasing SKP2 expression via HIF-1
Source: Oncogene. 2020 Aug 21;39(39):6218–30. doi: 10.1038/s41388-020-01423-8 (PMC7515798; doi:10.1038/s41388-020-01423-8)
Supplement: Supplementary file 6 — Supplementary Figure 5 [file 41388_2020_1423_MOESM6_ESM.pdf]

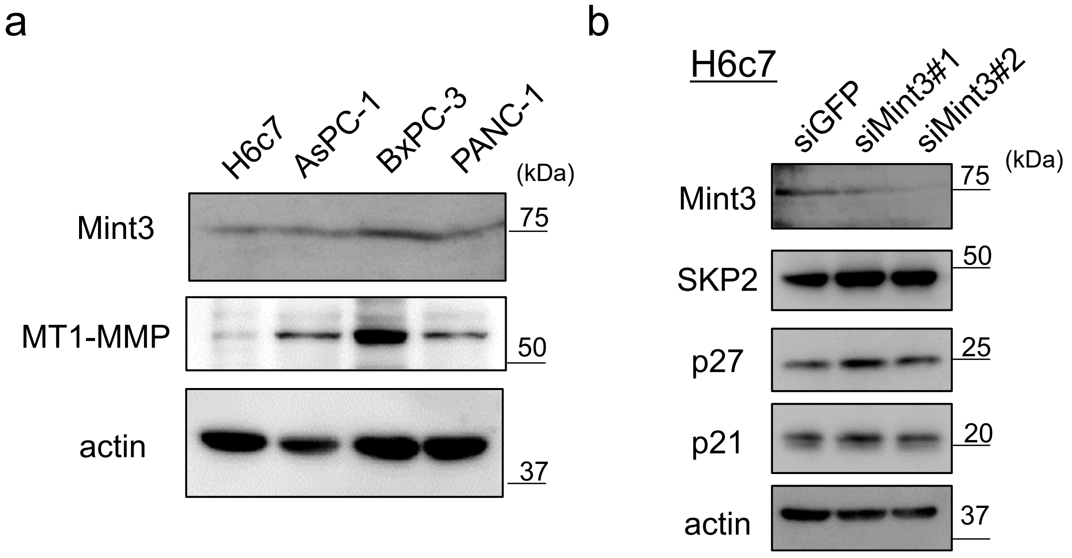

Supplementary Figure 5. Mint3 does not control SKP2 expression in pancreatic epithelial H6c7 cells.
(**a**) Immunoblot of Mint3, MT1-MMP, and actin in immortalized pancreatic duct epithelial H6c7 cells and pancreatic cancer AsPC-1, BxPC-3, and PANC-1 cells.
(**b**) Immunoblot of in H6c7 cells treated with control siRNA (siGFP) or Mint3 siRNA (siMint3#1, #2).
