# Supplementary material for: Mint3 depletion restricts tumor malignancy of pancreatic cancer cells by decreasing SKP2 expression via HIF-1
Source: Oncogene. 2020 Aug 21;39(39):6218–30. doi: 10.1038/s41388-020-01423-8 (PMC7515798; doi:10.1038/s41388-020-01423-8)
Supplement: Supplementary file 7 — Supplementary Figure 6 [file 41388_2020_1423_MOESM7_ESM.pdf]

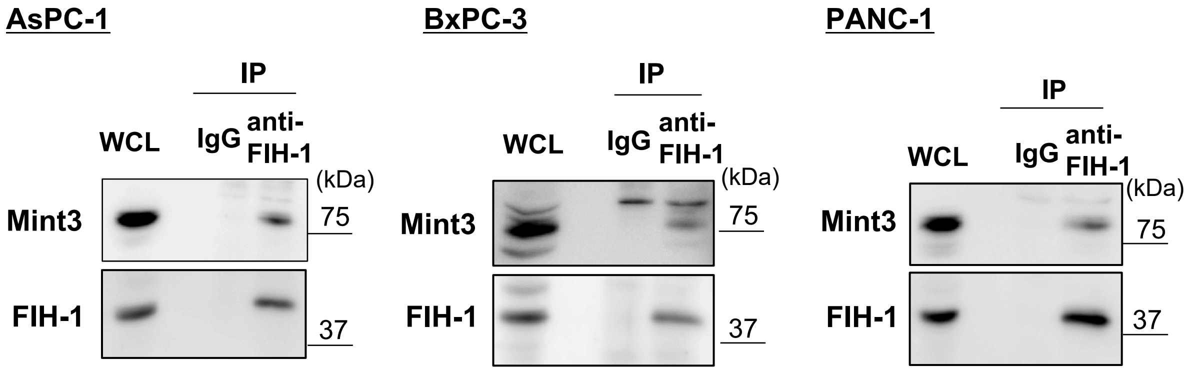

Supplementary Figure 6. Endogenous Mint3 binds to FIH-1 in pancreatic cancer cells.
FIH-1 in AsPC-1, BxPC-3, and PANC-1 cells was immunoprecipitated using control IgG or anti-FIH-1 antibodies, and co-precipitated Mint3 was detected by immunoblotting.
