# Supplementary material for: Mint3 depletion restricts tumor malignancy of pancreatic cancer cells by decreasing SKP2 expression via HIF-1
Source: Oncogene. 2020 Aug 21;39(39):6218–30. doi: 10.1038/s41388-020-01423-8 (PMC7515798; doi:10.1038/s41388-020-01423-8)
Supplement: Supplementary file 8 — Supplementary Figure 7 [file 41388_2020_1423_MOESM8_ESM.pdf]

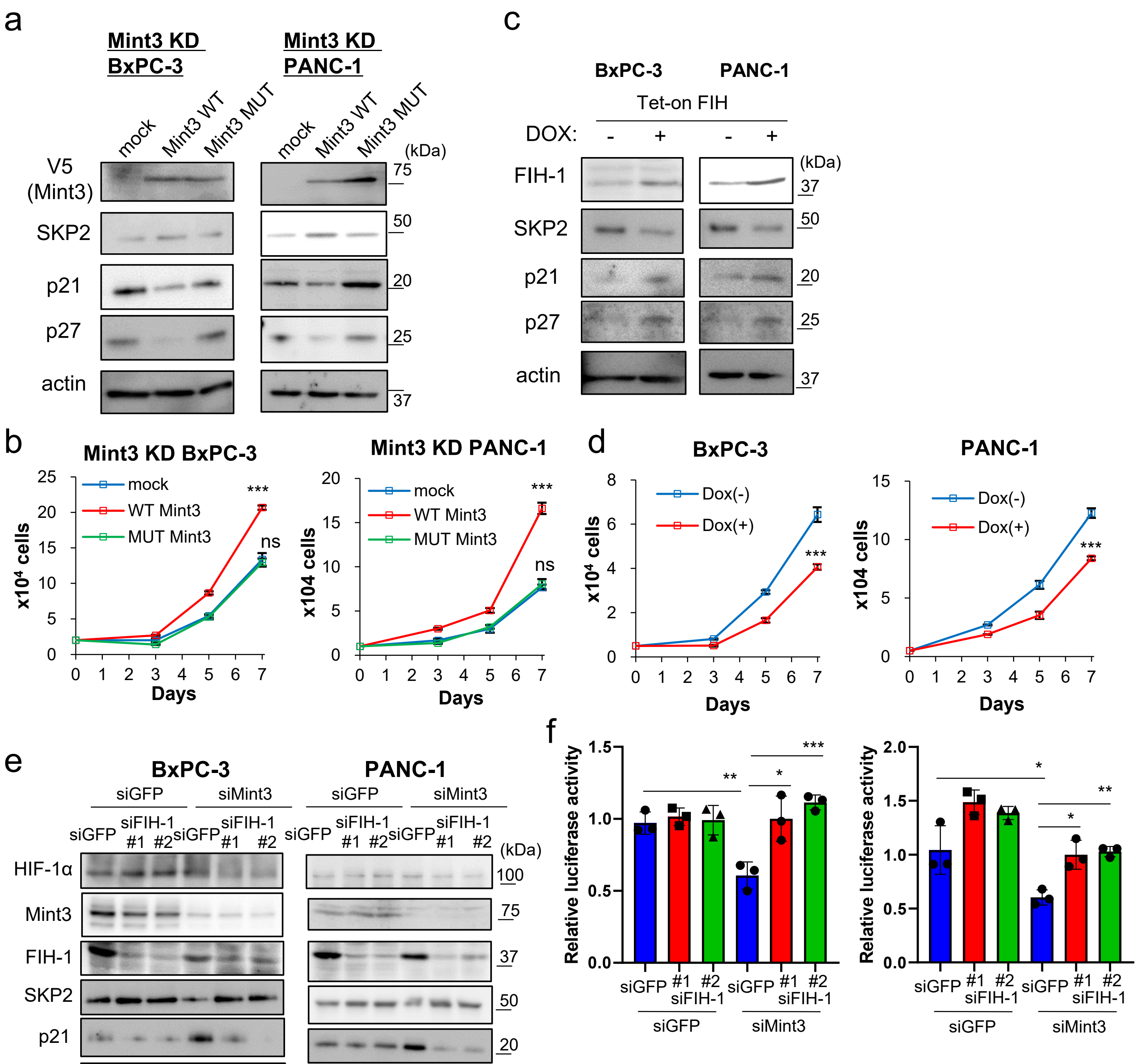

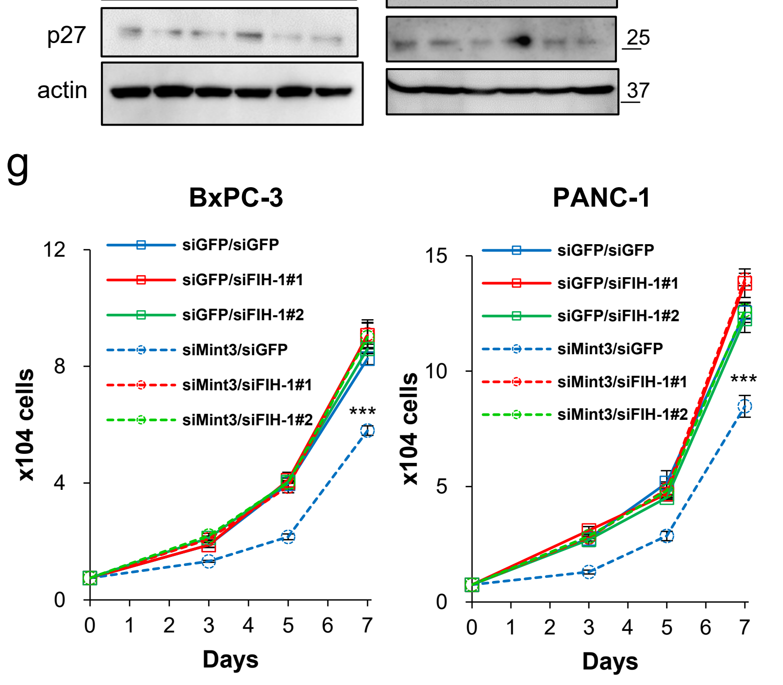

Supplementary Figure 7. The Mint3-FIH axis is involved in cell growth in pancreatic cancer cells.
(**a**) SKP2, p21, and p27 expression in Mint3-knockdown (KD) BxPC-3 and PANC-1 cells expressing mock, V5-tagged WT, or MUT Mint3.
(**b**) Cell growth assay of Mint3-KD BxPC-3 and PANC-1 cells expressing mock, V5-tagged WT, or MUT Mint3.
(**c**) SKP2, p21, and p27 expression in doxycycline (DOX)-induced FIH-1-expressing BxPC-3 and PANC-1 cells.
(**d**) Cell growth assay of DOX-induced FIH-1-expressing BxPC-3 and PANC-1 cells.
(**e–g**) Immunoblot analysis of BxPC-3 and PANC-1 cells treated with control siRNA (siGFP), Mint3 siRNA (siMint3), and FIH-1 siRNA (siFIH-1#1, #2) and HIF-1 activity (**f**) and cell growth assay of these cells (**g**).
Error bars indicate SD (n = 3). *$p < 0.05$, **$p < 0.01$, ***$p < 0.001$, ns, not significant (*t*-test).
