# Supplementary material for: Mint3 depletion restricts tumor malignancy of pancreatic cancer cells by decreasing SKP2 expression via HIF-1
Source: Oncogene. 2020 Aug 21;39(39):6218–30. doi: 10.1038/s41388-020-01423-8 (PMC7515798; doi:10.1038/s41388-020-01423-8)
Supplement: Supplementary file 9 — Supplementary Figure 8 [file 41388_2020_1423_MOESM9_ESM.pdf]

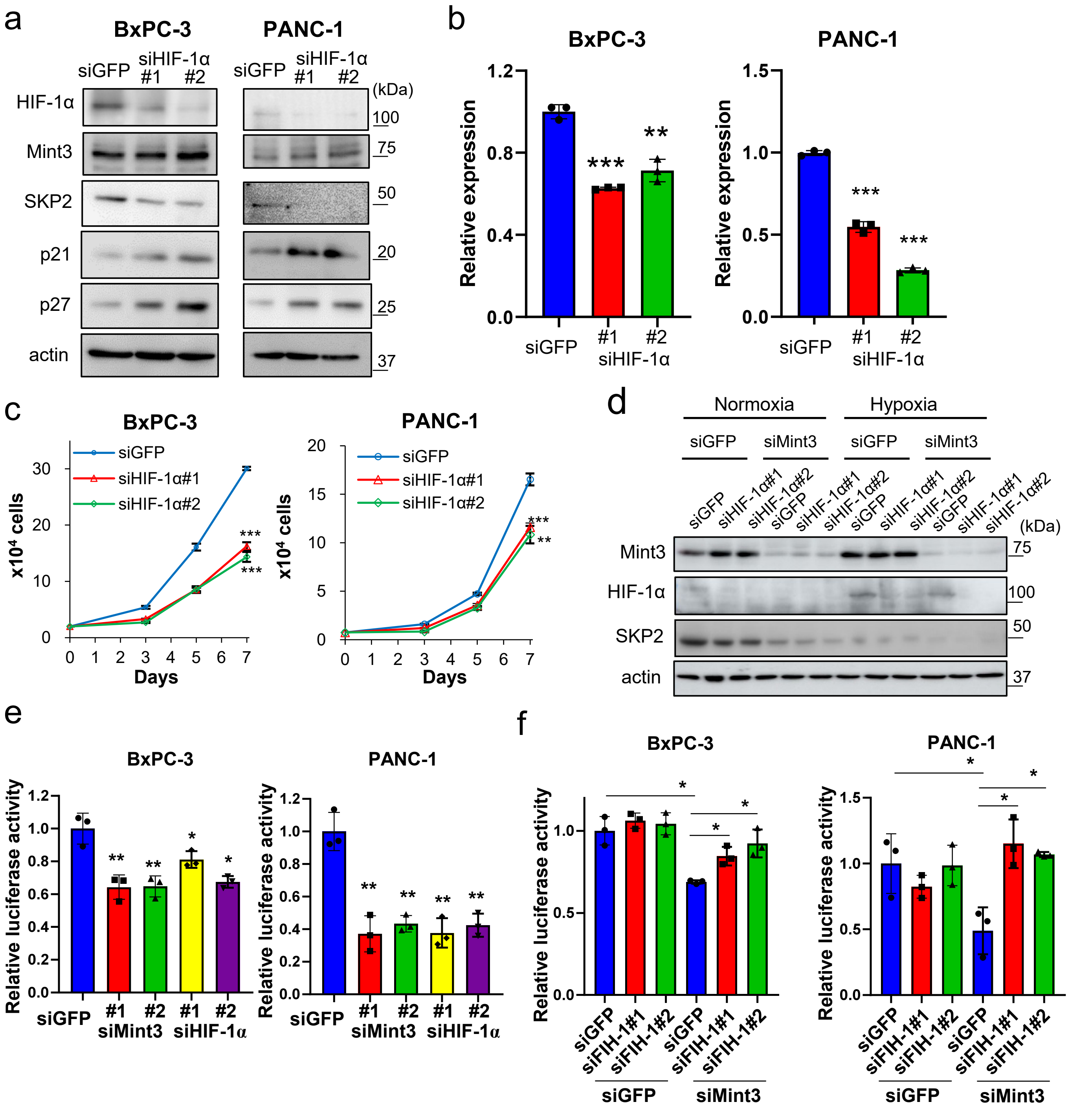

Supplementary Figure 8. HIF-1α depletion decreases SKP2 expression and cell growth in pancreatic cancer cells.

(**a**) SKP2, p21, and p27 expression in control (siGFP) and HIF-1α-depleted (siHIF-1α#1, #2) BxPC-3 and PANC-1 cells.

(**b**) SKP2 mRNA levels in BxPC-3 and PANC-1 cells transfected with control siRNA or HIF-1α siRNA. Expression levels were normalized to *ACTB*.

(**c, d**) Cell growth (c) and HIF-1α activity (**d**) of control and HIF-1α-depleted BxPC-3 and PANC-1 cells.

(**e**) Immunoblotting of HIF-1α, Mint3, and SKP2 in AsPC-1 cells transfected with siGFP, siMint3, and siHIF-1α during normoxia and hypoxia.

(**f**) SKP2 promoter activity in BxPC-3 and PANC-1 cells transfected with siGFP, siMint3, and siHIF-1α.

(**g**) SKP2 promoter activity in BxPC-3 and PANC-1 cells transfected with siGFP, siMint3, and siFIH-1.

Error bars indicate SD (n = 3). *$p < 0.05$, **$p < 0.01$, ***$p < 0.001$ (*t*-test).
