# Supplementary material for: Mint3 depletion restricts tumor malignancy of pancreatic cancer cells by decreasing SKP2 expression via HIF-1
Source: Oncogene. 2020 Aug 21;39(39):6218–30. doi: 10.1038/s41388-020-01423-8 (PMC7515798; doi:10.1038/s41388-020-01423-8)
Supplement: Supplementary file 10 — Supplementary Figure 9 [file 41388_2020_1423_MOESM10_ESM.pdf]

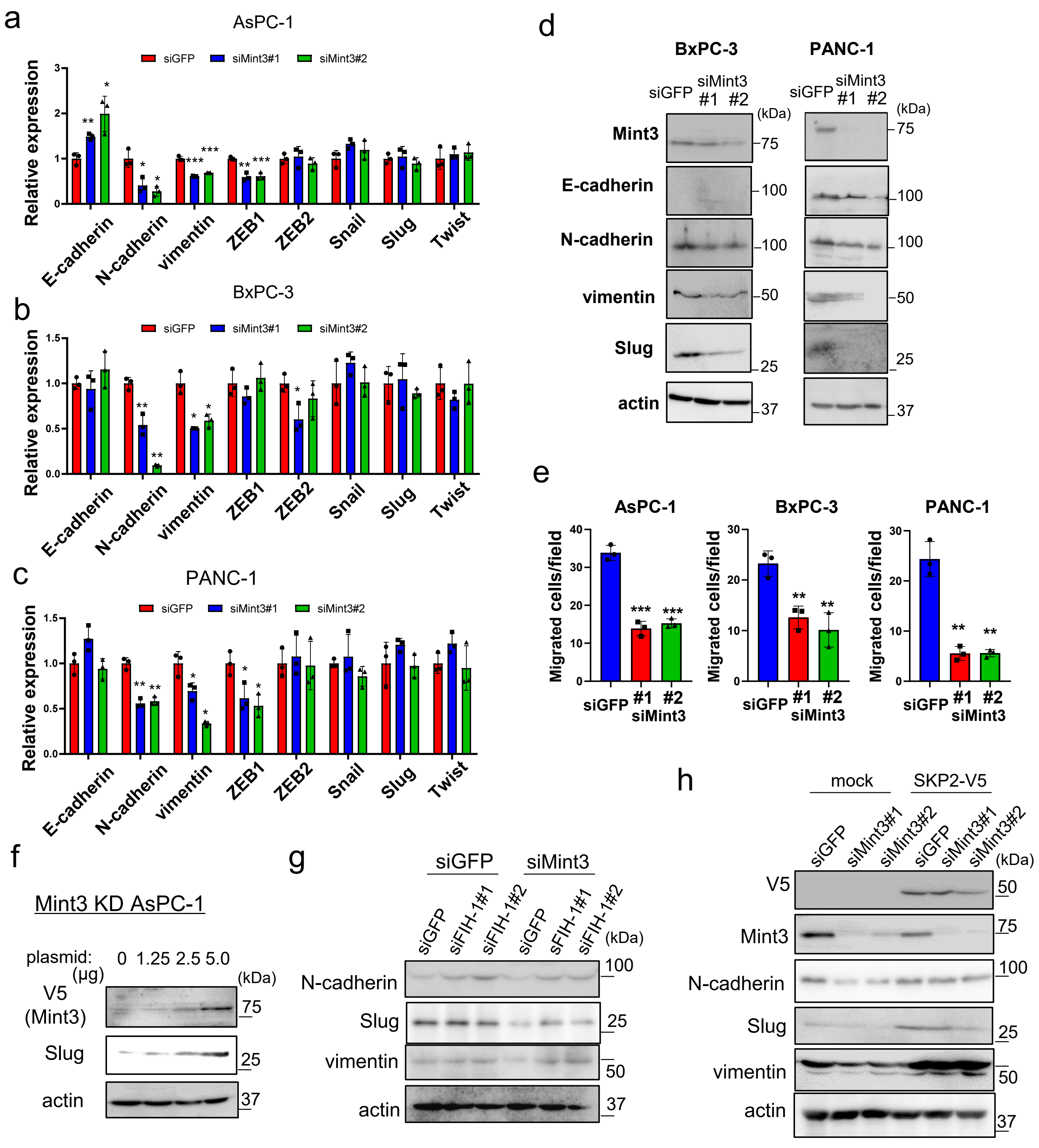

Supplementary Figure 9. Mint3 depletion attenuates mesenchymal features in pancreatic cancer cells.
(**a–c**) mRNA levels of EMT-related genes in AsPC-1 (**a**), BxPC-3 (**b**), and PANC-1 cells (**c**) transfected with control siRNA (siGFP) or Mint3 siRNA (siMint3#1, #2). Expression levels were normalized to *ACTB*.
(**d**) Immunoblotting of EMT-related proteins in control (siGFP) and Mint3-depleted (siMint3) BxPC-3 and PANC-1 cells.
(**e**) Transwell migration assays of control and Mint3-depleted AsPC-1, BxPC-3, and PANC-1 cells.
(**f**) V5-tagged Mint3 was transiently expressed in Mint3-depleted AsPC-1 cells at various levels and Slug expression was analyzed by immunoblotting.
(**g**) Immunoblotting of EMT-related proteins in control (siGFP), Mint3-depleted (siMint3), and FIH-1-depleted (siFIH-1) AsPC-1 cells.
(**h**) Immunoblotting of EMT-related proteins in control (siGFP) and Mint3-depleted (siMint3) AsPC-1 cells expressing mock or V5-tagged SKP2.
Error bars indicate SD (n = 3). *$p < 0.05$, **$p < 0.01$, ***$p < 0.001$ (*t*-test).
