# Supplementary material for: Mint3 depletion restricts tumor malignancy of pancreatic cancer cells by decreasing SKP2 expression via HIF-1
Source: Oncogene. 2020 Aug 21;39(39):6218–30. doi: 10.1038/s41388-020-01423-8 (PMC7515798; doi:10.1038/s41388-020-01423-8)
Supplement: Supplementary file 11 — Supplementary Figure 10 [file 41388_2020_1423_MOESM11_ESM.pdf]

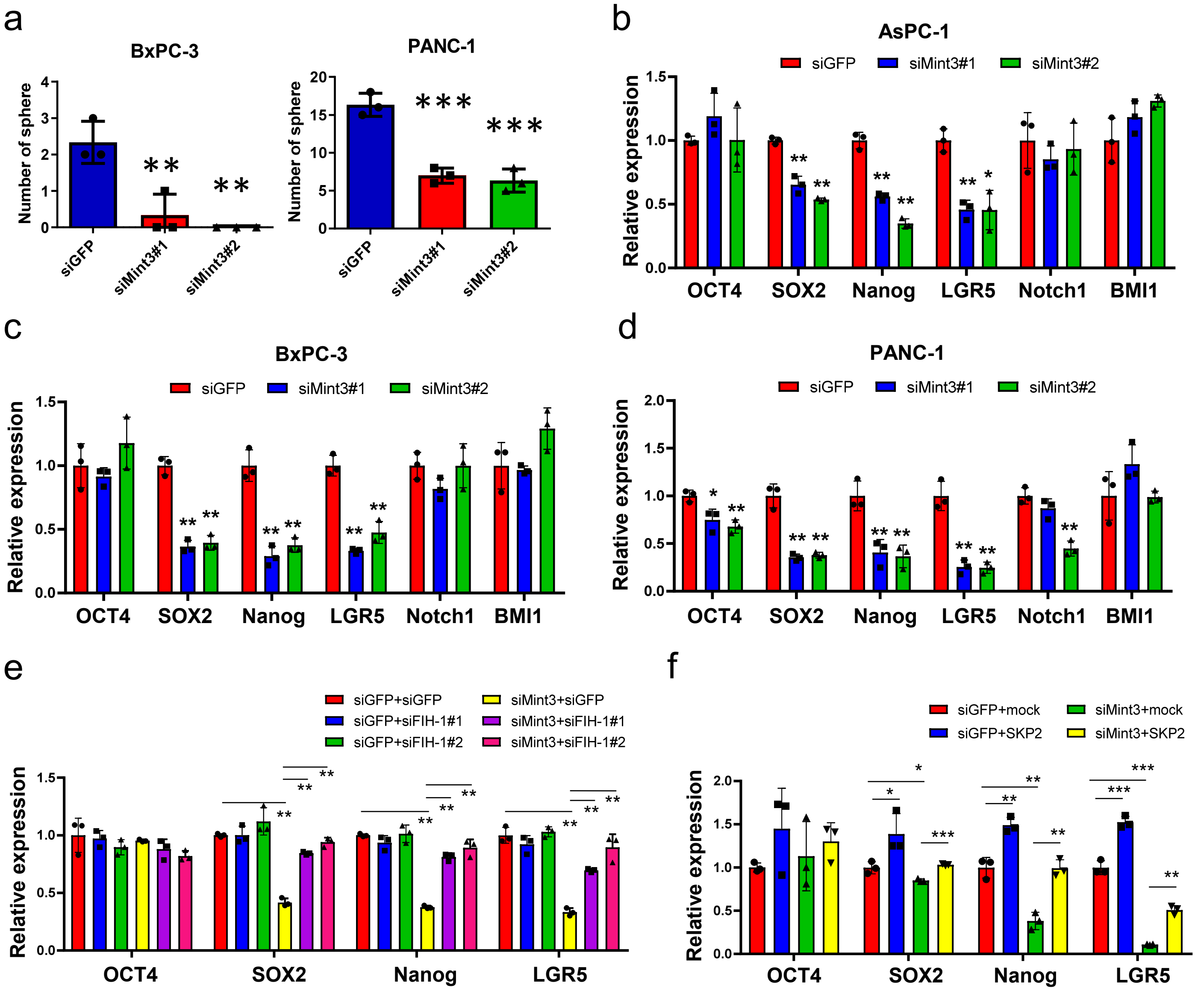

Supplementary Figure 10. Mint3 controls stemness in a FIH-1- and SKP2-dependent manner.
(**a**) Sphere formation assay of control (siGFP) and Mint3-depleted (siMint3) PANC-1 and BxPC-3 cells.
(**b–d**) mRNA levels of stemness marker genes in AsPC-1 (**b**), BxPC-3 (**c**), and PANC-1 cells (**d**) transfected with control siRNA (siGFP) or Mint3 siRNA (siMint3#1, #2).
(**e**) mRNA levels of stemness-related genes in control (siGFP), Mint3-depleted (siMint3), and FIH-1-depleted (siFIH-1) AsPC-1 cells.
(**f**) mRNA levels of stemness-related genes in control (siGFP) and Mint3-depleted (siMint3) AsPC-1 cells expressing mock or V5-tagged SKP2.
Expression levels were normalized to *ACTB*. Error bars indicate SD (n = 3). *$p < 0.05$, **$p < 0.01$, ***$p < 0.001$, ns, not significant (*t*-test).
