# Supplementary material for: Mint3 depletion restricts tumor malignancy of pancreatic cancer cells by decreasing SKP2 expression via HIF-1
Source: Oncogene. 2020 Aug 21;39(39):6218–30. doi: 10.1038/s41388-020-01423-8 (PMC7515798; doi:10.1038/s41388-020-01423-8)
Supplement: Supplementary file 12 — Supplementary Figure 11 [file 41388_2020_1423_MOESM12_ESM.pdf]

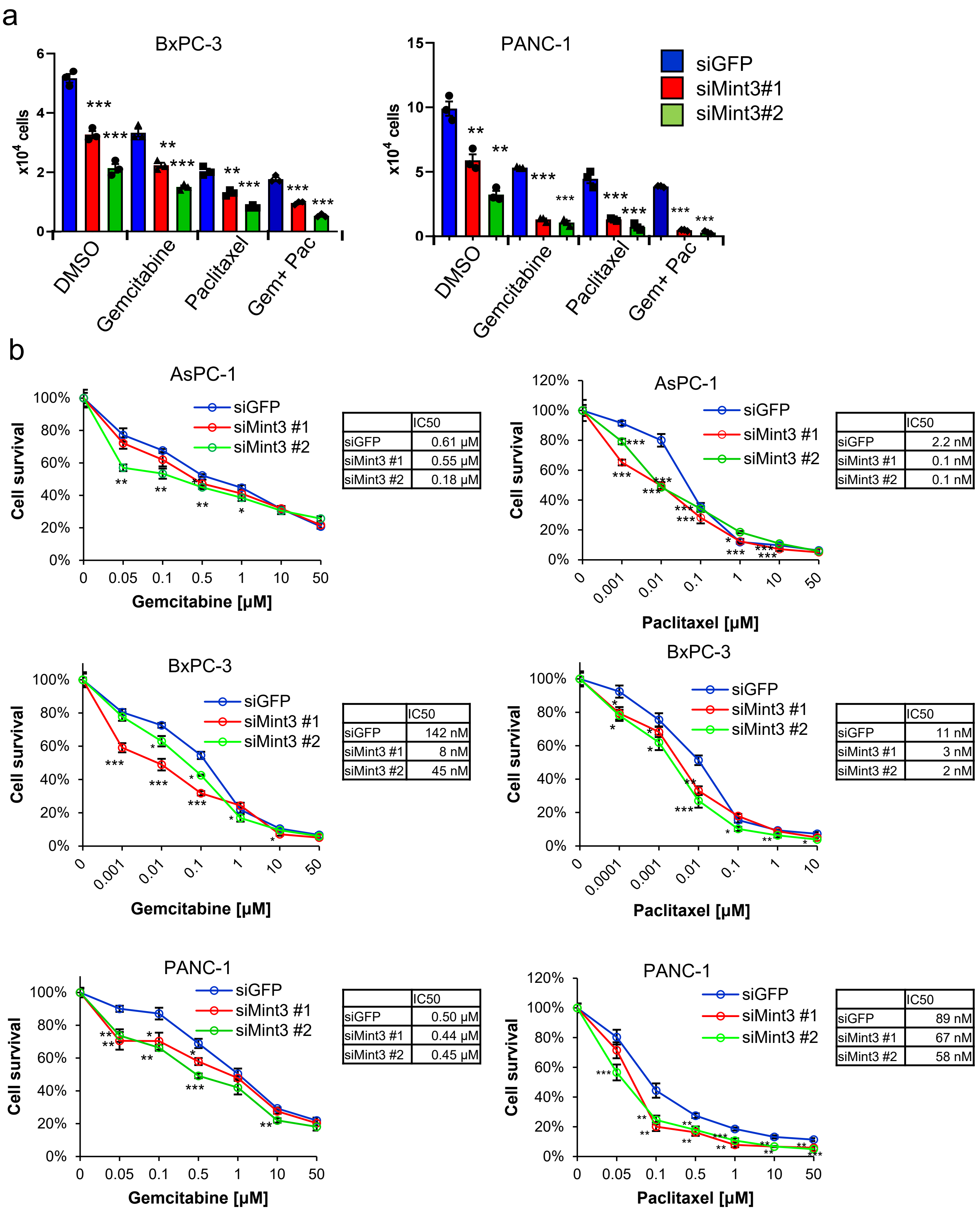

Supplementary Figure 11. Mint3 depletion attenuates chemoresistance in BxPC-3 and PANC-1 cells.
(**a**) Control (siGFP) and Mint3-depleted (siMint3) BxPC-3 (left) and PANC-1 cells (right) were counted after treatment with DMSO, gemcitabine (50 nM for BxPC-3 and 50 μM for PANC-1), paclitaxel (5 nM for BxPC-3 and 50 μM for PANC-1), or both gemcitabine and paclitaxel for 3 days.
(**b**) Cell survival of pancreatic cancer cells treated with different concentrations of gemcitabine or paclitaxel. Cells were counted after treatment with DMSO, gemcitabine, or paclitaxel for 3 days.
Error bars indicate SD (n = 3). *$p < 0.05$, **$p < 0.01$, ***$p < 0.001$ (t-test).
