# Supplementary material for: Mint3 depletion restricts tumor malignancy of pancreatic cancer cells by decreasing SKP2 expression via HIF-1
Source: Oncogene. 2020 Aug 21;39(39):6218–30. doi: 10.1038/s41388-020-01423-8 (PMC7515798; doi:10.1038/s41388-020-01423-8)
Supplement: Supplementary file 13 — Supplementary Figure 12 [file 41388_2020_1423_MOESM13_ESM.pdf]

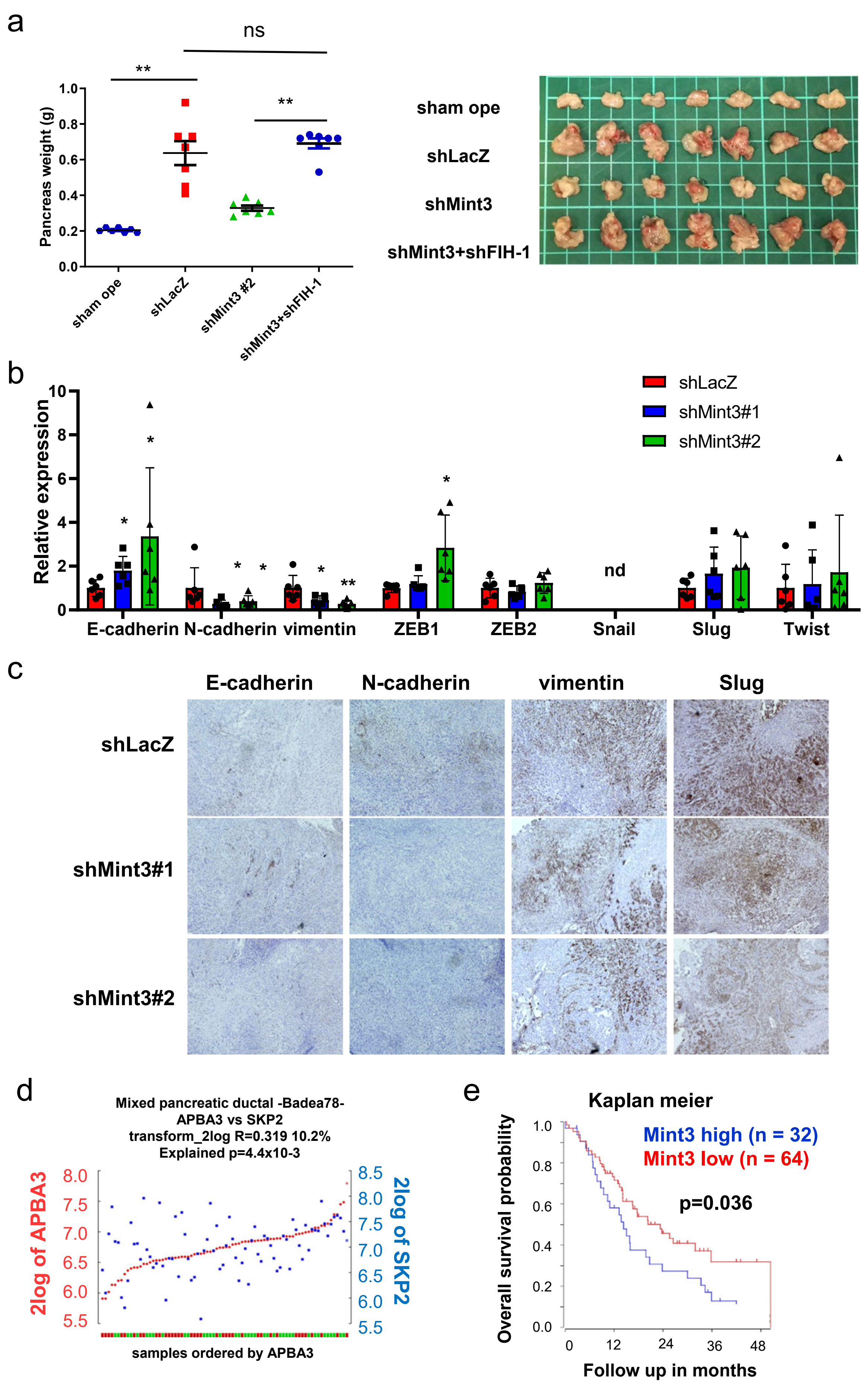
a
ns
**
**
Pancreas weight (g)
1.0
0.8
0.6
0.4
0.2
0.0
sham ope
shLacZ
shMint3 #2
shMint3+shFIH-1
sham ope
shLacZ
shMint3
shMint3+shFIH-1
b
Relative expression
shLacZ
shMint3#1
shMint3#2
nd
E-cadherin
N-cadherin
vimentin
ZEB1
ZEB2
Snail
Slug
Twist
c
E-cadherin
N-cadherin
vimentin
Slug
shLacZ
shMint3#1
shMint3#2
d
Mixed pancreatic ductal -Badea78-
APBA3 vs SKP2
transform_2log R=0.319 10.2%
Explained p=4.4x10-3
2log of APBA3
2log of SKP2
samples ordered by APBA3
e
Kaplan meier
Overall survival probability
Mint3 high (n = 32)
Mint3 low (n = 64)
p=0.036
Follow up in months

Supplementary Figure 12. Mint3 promotes tumor malignancy of pancreatic cancer.
(**a**) Tumor growth of orthotopically inoculated control (shLacZ), Mint3-depleted (shMint3), or both Mint3 and FIH-1 depleted (shMint3 + shFIH-1) AsPC-1 cells in the pancreas of immunodeficient mice (n = 7). Error bars indicate SD. $^{**}p < 0.01$ (Mann-Whitney U test).
(**b**) mRNA levels of EMT-related genes in pancreatic tumors of control and Mint3-depleted AsPC-1 cells. Expression levels were normalized to *ACTB*. Error bars indicate SD (n = 6 per group). $^{*}p < 0.05$, $^{**}p < 0.01$ (Mann-Whitney U test). nd, not detected.
(**c**) Immunohistochemical analysis of E-cadherin, N-cadherin, Slug, and vimentin in tumors of control (shLacZ) and Mint3-depleted (shMint3) AsPC-1 cells.
(**d**) *APBA3* (encoding Mint3) and *SKP2* mRNA levels in human pancreatic cancer from the Badea 78 dataset of The Cancer Genome Atlas database. The correlation factor was R = 0.319, and $p = 4.4 \times 10^{-3}$ (one-way ANOVA).
(**e**) Kaplan-Meier plot analysis of overall survival of pancreatic cancer patients with Mint3 high (n = 32, blue) and low (n = 64, red) expression from the Bailey 96 dataset of R2 database. $p = 0.036$ (log-rank test).
