# Supplementary figures and images for: Mint3 depletion restricts tumor malignancy of pancreatic cancer cells by decreasing SKP2 expression via HIF-1
Source: Oncogene. 2020 Aug 21;39(39):6218–30. doi: 10.1038/s41388-020-01423-8 (PMC7515798; doi:10.1038/s41388-020-01423-8)

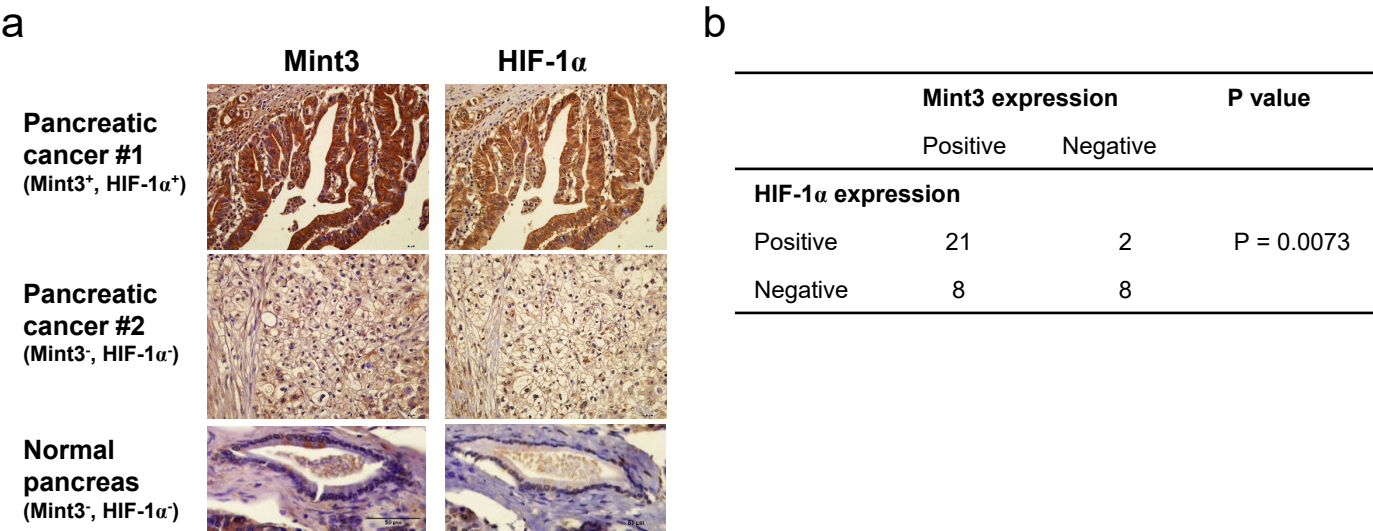

Supplement: Supplementary file 14 — Supplementary Figure 13 [file 41388_2020_1423_MOESM14_ESM.pdf]
