# Supplementary material for: Mint3 depletion restricts tumor malignancy of pancreatic cancer cells by decreasing SKP2 expression via HIF-1
Source: Oncogene. 2020 Aug 21;39(39):6218–30. doi: 10.1038/s41388-020-01423-8 (PMC7515798; doi:10.1038/s41388-020-01423-8)
Supplement: Supplementary file 15 — Supplementary Figure 14 [file 41388_2020_1423_MOESM15_ESM.pdf]

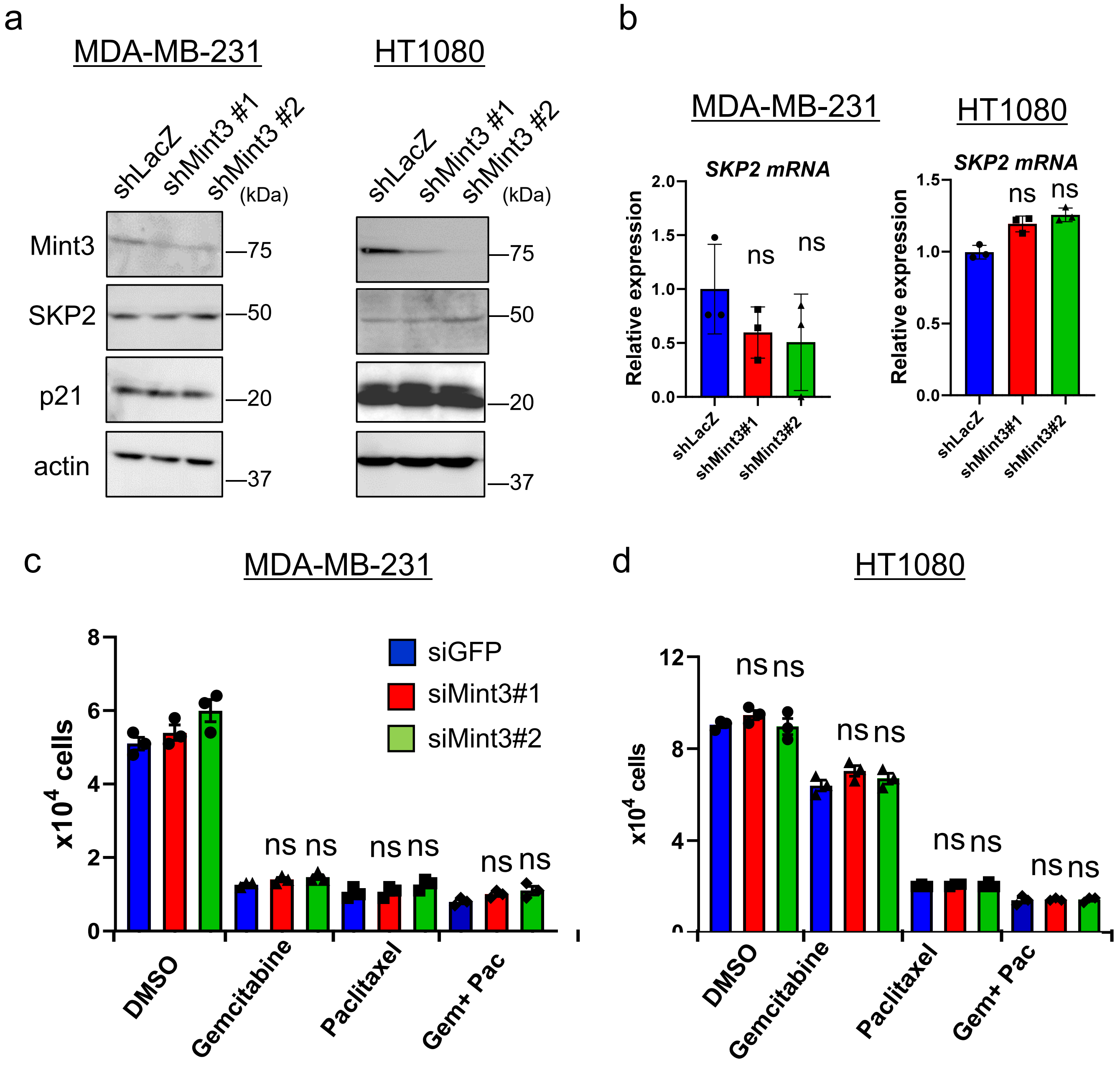

Supplementary Figure 14. Mint3 does not control SKP2 expression in non-pancreatic cancer cells.
(**a**) Expression of Mint3, SKP2, p21, and actin in control (shLacZ) and Mint3-depleted (shMint3) MDA-MB-231 (left) and HT1080 cells (right).
(**b**) *SKP2* mRNA levels in control and Mint3-depleted MDA-MB-231 (left) and HT1080 cells (right). Expression levels were normalized to *ACTB*.
(**c**, **d**) Control and Mint3-depleted MDA-MB-231 (**c**) and HT1080 cells (**d**) were counted after treatment with DMSO, gemcitabine (50 mM), paclitaxel (50 mM), or both gemcitabine and paclitaxel for 3 days.
Error bars indicate SD (n = 3). ns, not significant (*t*-test).
