# Supplementary material for: Mint3 depletion restricts tumor malignancy of pancreatic cancer cells by decreasing SKP2 expression via HIF-1
Source: Oncogene. 2020 Aug 21;39(39):6218–30. doi: 10.1038/s41388-020-01423-8 (PMC7515798; doi:10.1038/s41388-020-01423-8)
Supplement: Supplementary file 16 — Supplementary Figure 15 [file 41388_2020_1423_MOESM16_ESM.pdf]

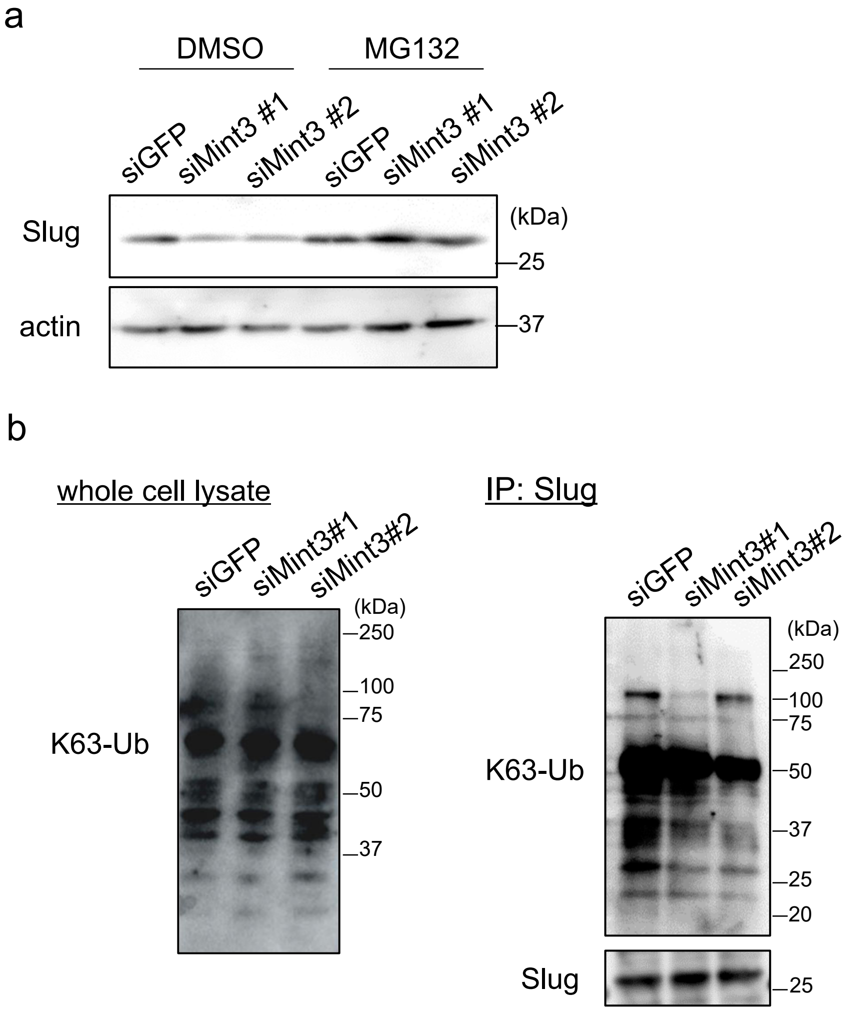

Supplementary Figure 15. Mint3 depletion decreases Slug protein levels in a proteasomal degradation dependent manner in AsPC-1 cells.

(**a**) AsPC-1 cells were transfected with siRNAs against GFP (siGFP) or Mint3 (siMint3). Five days later, cells were treated with DMSO or MG132 (10 mM) for 4 h prior to harvesting cells.

(**b**) Slug protein in control (siGFP) and Mint3-depleted (siMint3#1, #2) AsPC-1 cells were immunoprecipitated and detected using K63-linkage specific polyubiquitin antibody. Note that the K63-linkage polyubiquitination levels of Slug protein were decreased in Mint3-depleted AsPC-1 cells.
