# Supplementary material for: Mint3 depletion restricts tumor malignancy of pancreatic cancer cells by decreasing SKP2 expression via HIF-1
Source: Oncogene. 2020 Aug 21;39(39):6218–30. doi: 10.1038/s41388-020-01423-8 (PMC7515798; doi:10.1038/s41388-020-01423-8)
Supplement: Supplementary file 17 — Supplementary Table 1 [file 41388_2020_1423_MOESM17_ESM.docx]

|  | Sequence |
| --- | --- |
| shLacZ | 5’- GCTACACAAATCAGCGATTTCGAAAAATCGCTGATTTGTGTAG-3’ |
| shMint3 #1 | 5’- CCGACTGTTGCAGCCCCCTGACGAATCAGGGGGCTGCAACAGTCGG-3’ |
| shMint3 #2 | 5’- GCGGTTCTTGGTCCTGTATGACGAATCATACAGGACCAAGAACCGC -3’ |

**Supplementary Table 1.** shRNA sequences
