# Supplementary material for: Mint3 depletion restricts tumor malignancy of pancreatic cancer cells by decreasing SKP2 expression via HIF-1
Source: Oncogene. 2020 Aug 21;39(39):6218–30. doi: 10.1038/s41388-020-01423-8 (PMC7515798; doi:10.1038/s41388-020-01423-8)
Supplement: Supplementary file 18 — Supplementary Table 2 [file 41388_2020_1423_MOESM18_ESM.docx]

|  | Sequence |
| --- | --- |
| siLuc | 5’-GAUCUACUGGUCUGCCUAA-3’ |
| siGFP | 5’-AUCCGCGCGAUAGUACGUA -3’ |
| siMint3 #1 | 5’-CUGCAUACCAUCUCCUACA-3’ |
| siMint3 #2 | 5’-CCUAUGGCGAGGUGCAUAU-3’ |
| siMint3 #3 | 5’-GGUUCUUGGUCCUGUAUGA-3’ |
| siFIH-1 #1 | 5’-GCCAAUUUCCAGAACUUUA-3’ |
| siFIH-1 #2 | 5’-GUAUUGCACGCUGCACUUA-3’ |
| siHIF-1α#1 | 5’-CAAAGUUCACCUGAGCCU-3’ |
| siHIF-1α#2 | 5’-GAUUAACUCAGUUUGAACU-3’ |
| siSKP2 #1 | 5’-GACCUAUCGAACUCAGUUA-3’ |
| siSKP2 #2 | 5’-GUGAUUGUCCGCAGGCCUA-3’ |

**Supplementary Table 2.** siRNA sequences
