# Supplementary material for: Mint3 depletion restricts tumor malignancy of pancreatic cancer cells by decreasing SKP2 expression via HIF-1
Source: Oncogene. 2020 Aug 21;39(39):6218–30. doi: 10.1038/s41388-020-01423-8 (PMC7515798; doi:10.1038/s41388-020-01423-8)
Supplement: Supplementary file 19 — Supplementary Table 3 [file 41388_2020_1423_MOESM19_ESM.docx]

| Antibody | Dilution |
| --- | --- |
| Anti-Skp2 (D3G5) rabbit mAb (CST, 2652) | 1/1000 |
| Anti-Mint3 mouse antibody (BD Biosciences, 611380) | 1/1000 |
| Anti-HIF-1α (BD Biosciences, 610598) | 1/1000 |
| Anti-Caspase-3 rabbit antibody (CST, 9664) | 1/500 |
| Anti-Cleaved rabbit caspase-3 antibody (CST, 9665) | 1/500 |
| Anti-PARP rabbit antibody (CST, 9542) | 1/500 |
| Anti-Cleaved PARP rabbit antibody (CST, 5625) | 1/500 |
| Anti-Caspase-9 mouse antibody (CST, 9508) | 1/500 |
| Anti-Cleaved caspase-9 rabbit antibody (CST, 7237) | 1/500 |
| Anti-Caspase-7 rabbit antibody (CST, 12827) | 1/500 |
| Anti-Cleaved caspase-7 rabbit antibody (CST, 8434) | 1/500 |
| Anti-Phospho histon H3 rabbit antibody (CST, 3377) | 1/1000 |
| Anti-Cyclin D1 (92G2) rabbit antibody (CST, 2978) | 1/500 |
| Anti-CyclinE2 rabbit antibody (CST, 4132) | 1/500 |
| Anti-CDK4 (D9G3E) rabbit antibody (CST, 12790) | 1/500 |
| Anti-CDK6 (DCS83) mouse antibody (CST, 3136) | 1/500 |
| Anti-p21 rabbit antibody (CST, 2947P) | 1/500 |
| Anti-p27 rabbit antibody (CST, 3686) | 1/500 |
| Anti-PCNA antibody (Santa Cruz, sc-56) | 1/1000 |
| Anti-FIH-1 Goat antibody (Santa Cruz, Sc-26219) | 1/500 |
| Anti-β-Actin Mouse antibody (FUJIFILM Wako, 0111-24554) | 1/1000 |
| Anti-E-cadherin (BD Biosciences, #610181) | 1/1000 |
| Anti-N-cadherin rabbit antibody (CST, 13316) | 1/1000 |
| Anti-Slug rabbit (CST, 9585) | 1/1000 |
| Anti-TWIST (Novus, NBP2-37364) | 1/1000 |
| Anti-Vimentin rabbit antibody (CST, 5741) | 1/1000 |
| Anti-Snail rabbit antibody (CST, 3879) | 1/500 |
| Anti-TCF8/ZEB1 rabbit antibody (CST, 3396) | 1/1000 |
| Anti-K48-linkage Specific Polyubiquitin Antibody (CST, #8081) | 1/1000 |
| Anti-K63-linkage specific polyubiquitin antibody (CST, #5621) | 1/1000 |
| Anti-V5 mouse antibody (Thermo Fisher Scientific, R960-25) | 1/1000 |
| Anti-MT1-MMP rabbit antibody (Millipore, AB6004) | 1/1000 |
| Anti-rabbit IgG, HRP linked whole Ab (GE Helthcare, NA934V) | 1/3000 |
| Anti-mouse IgG, HRP- linked whole Ab (GE Helthcare, NA931V) | 1/3000 |
| Anti-Goat IgG HRP antibody (Sigma, A5420) | 1/5000 |

**Supplementary Table 3.** Antibodies used in western blotting
