# Supplementary material for: Mint3 depletion restricts tumor malignancy of pancreatic cancer cells by decreasing SKP2 expression via HIF-1
Source: Oncogene. 2020 Aug 21;39(39):6218–30. doi: 10.1038/s41388-020-01423-8 (PMC7515798; doi:10.1038/s41388-020-01423-8)
Supplement: Supplementary file 20 — Supplementary Table 4 [file 41388_2020_1423_MOESM20_ESM.docx]

| Gene name | Forward primer | Reverse Primer |
| --- | --- | --- |
| ACTB  SKP2  CDKN1A (p21)  CDKN1B (p27)  HIF-1α  VEGFA  PDK1  E-cadherin  N-cadherin  vimentin  ZEB1  ZEB2  Snail  Slug  Twist  OCT4  SOX2  Nanog  LGR5  Notch1  BMI | TTCTACAATGAGCTGCGTGTG  CTTTACTATTAGTGACAAGAGCTGG  TCAGGGGAGCAGGCTGAA  GCTAACTCTGAGGACACGCA  ATCCATGTGACCATGAGGAAATG  CTCCACCATGCCAAGTGGTC  TCCTGTCACCAGCCAGAATG  CAATGCCGCCATCGCTTACACCAT  AGGCTTCTGGTGAAATCGCA  GACGCCATCAACACCGAGTT  GGGCCTGAAGCTCAGGCAGATGA  AGAAGCCACGATCCAGACCGCAATTA  CCAGTGCCTCGACCACTATG  ATGCATATTCGGACCCACACATTA  TCCATGTCCGCGTCCCACTA  GGGGTTCTATTTGGGAAGGTAT  GAACCAGCGCATGGACAGTT  GGTGGAGTATGGTTGGAGCC  CCTGCTTGACTTTGAGGAAGACC  CCCGCCAGAGTGGACAGGTCAGTA  CTGGTTGCCCATTGACAGCG | GGGGTGTTGAAGGTCTCAAA  TGGCTGGACTTGAGTTTGGA  TGGTAGAAATCTGTCATGCTGGT  GAAGAATCGTCGGTTGCAGGT  CTCGGCTAGTTAGGGTACACTT  ACTCCTGGAAGATGTCCACC  CTTCCTTTGCCTTTTCCACC  TCAGCAGCTTGAACCACCAGGGTA  TGCAGTTGCTAAACTTCACATTG  CTTTGTCGTTGGTTAGCTGGT  CTCTGGTCCTCTTCAGGTGCCTC  GGTAAATAATGGCTGTGTCACTGCGC  CTGCTGGAAGGTAAACTCTGGA  AGATTTGACCTGTCTGCAAATGCTC  ATTCAAAGAAACAGGGCGTG  GCCGCAGCTTACACATGTTC  TCATGCTGTAGCTGCCGTTG  GAGACGGCAGCCAAGGTTAT  CCAGCCATCAAGCAGGTGTTCA  TGTCGCAGTTGGAGCCCTCGTTA  AAATCCCGGAAAGAGCAGCC |

**Supplementary Table 4.** Primer pairs used in quantitative real-time PCR
