# Supplementary material for: Mint3 depletion restricts tumor malignancy of pancreatic cancer cells by decreasing SKP2 expression via HIF-1
Source: Oncogene. 2020 Aug 21;39(39):6218–30. doi: 10.1038/s41388-020-01423-8 (PMC7515798; doi:10.1038/s41388-020-01423-8)
Supplement: Supplementary file 21 — Supplementary Table 5 [file 41388_2020_1423_MOESM21_ESM.docx]

| Antibody | Dilution |
| --- | --- |
| Anti-SKP2 Rabbit antibody (CST, 2652) | 1/100 |
| Anti-Mint3 mouse antibody (BD Biosciences, 611380) | 1/50 |
| Anti-HIF-1α antibody (Novus, NB100-479) | 1/50 |
| Anti-p21 rabbit antibody (CST, 2947P) | 1/100 |
| Anti-p27 rabbit antibody (CST, 3686) | 1/100 |
| Anti-E-cadherin (BD, 610181) | 1/100 |
| Anti-N-cadherin rabbit antibody (CST, 13316) | 1/100 |
| Anti-Slug mouse antibody (Santa cruz, sc-166476) | 1/50 |
| Anti-TWIST1 mouse antibody (Novus, NBP-237364) | 1/200 |
| Anti-Vimentin rabbit antibody (CST, 5741) | 1/100 |

**Supplementary Table 5.** Antibodies used in immunostaining of paraffin-embedded sections
