# Supplementary material for: Mint3 depletion restricts tumor malignancy of pancreatic cancer cells by decreasing SKP2 expression via HIF-1
Source: Oncogene. 2020 Aug 21;39(39):6218–30. doi: 10.1038/s41388-020-01423-8 (PMC7515798; doi:10.1038/s41388-020-01423-8)
Supplement: Supplementary file 22 — Supplementary Table 6 [file 41388_2020_1423_MOESM22_ESM.docx]

| Antibody | Dilution |
| --- | --- |
| HIF-1α antibody (Novus, NB100-479) | 1/50 |
| Anti Hypoxyprobe-1 (HPI, Burlington, MA, HP1-100) | 1/50 |
| Anti-Ki67 Antibody (Thermo Fisher Scientific, RM-9106-SO) | 1/50 |
| Goat anti-Rabbit IgG (H+L) Highly Cross-Adsorbed Secondary Antibody, Alexa Fluor 546 (Thermo Fisher Scientific, A11035) | 1/500 |
| Goat anti-Mouse IgG (H+L) Highly Cross-Adsorbed Secondary Antibody, Alexa Fluor 488 (Thermo Fisher Scientific, A11029) | 1/500 |

**Supplementary Table 6.** Antibodies used in immunostaining of frozen sections
